# Understanding Biomineralization Mechanisms to Produce Size-Controlled, Tailored Nanocrystals for Optoelectronic and Catalytic Applications: A Review

**DOI:** 10.1021/acsanm.3c04277

**Published:** 2024-02-29

**Authors:** Toriana N. Vigil, Leah C. Spangler

**Affiliations:** † University of Virginia, Charlottesville, Virginia 22903, United States; ‡ Virginia Commonwealth University, Richmond, Virginia 23284, United States

**Keywords:** Protein Engineering, Biomineralization, Quantum Dots, Nanoparticles, Energy, Templating

## Abstract

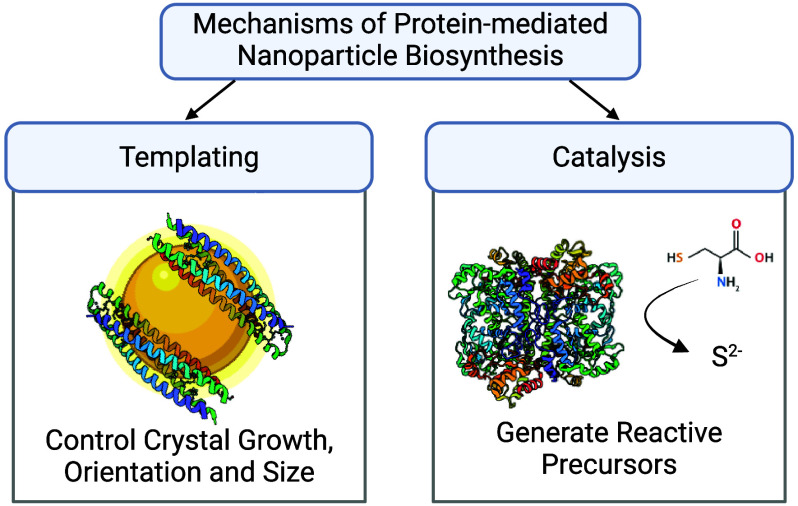

Biomineralization, the use of biological systems to produce inorganic materials, has recently become an attractive approach for the sustainable manufacturing of functional nanomaterials. Relying on proteins or other biomolecules, biomineralization occurs under ambient temperatures and pressures, which presents an easily scalable, economical, and environmentally friendly method for nanoparticle synthesis. Biomineralized nanocrystals are quickly approaching a quality applicable for catalytic and optoelectronic applications, replacing materials synthesized using expensive traditional routes. Here, we review the current state of development for producing functional nanocrystals using biomineralization and distill the wide variety of biosynthetic pathways into two main approaches: templating and catalysis. Throughout, we compare and contrast biomineralization and traditional syntheses, highlighting optimizations from traditional syntheses that can be implemented to improve biomineralized nanocrystal properties such as size and morphology, making them competitive with chemically synthesized state-of-the-art functional nanomaterials.

## Introduction

Biomineralization, by definition, is the process living organisms use to create inorganic materials for a biological purpose.^1^ Typically, living organisms employ biomineralization for protection, structural support, and directional orientation; examples include seashells, bones, and magnetic particles.^2^ Biomineralization can also be utilized outside of its original context and applied for the synthesis of functional materials, ranging from metal nanoparticle catalysts to semiconductor quantum dots.^3−7^ The synthetic production of nanoparticles as inspired by biomineralization is sometimes known as biomimetic,^8−16^ biodirected,^17−19^ biofabrication,^20^ biotransformation,^21^ bioprecipitation,^22^ or biogenic^23−26^ particle syntheses; however, broadly the term biomineralization remains common. Because biomineralization typically takes place in living organisms, the biological pathways used for functional nanomaterial synthesis occur under ambient temperatures and pressures in the aqueous phase. These conditions stand in stark contrast to the traditional methods of functional material synthesis that use organic solvents, high temperatures/pressures, and specialized equipment. Thus, biomineralization provides a scalable and sustainable method for the synthesis of functional inorganic materials.

To identify biomineralization pathways that can be used for functional nanomaterial synthesis, researchers often turn to systems found in nature that already produce the desired material and capitalize on the underlying pathway to synthesize related materials. For example, sea sponges produce highly ordered SiO_2_ structures using the enzyme silicatein that catalyzes the formation of transition metal oxides from water.^27^ Recently, silicatein has been used by several groups *in vitro* to direct SiO_2_ formation, synthesize Au nanoparticles, and produce catalytically active Ce_2_O nanoparticles in water at room temperature.^28−30^ By identifying the underlying enzyme responsible for mineralization, the exact protein can be isolated and recombinantly overexpressed to produce functional materials that are relevant for energy or catalysis applications without the complexities of the native organism.

**Figure 1:**
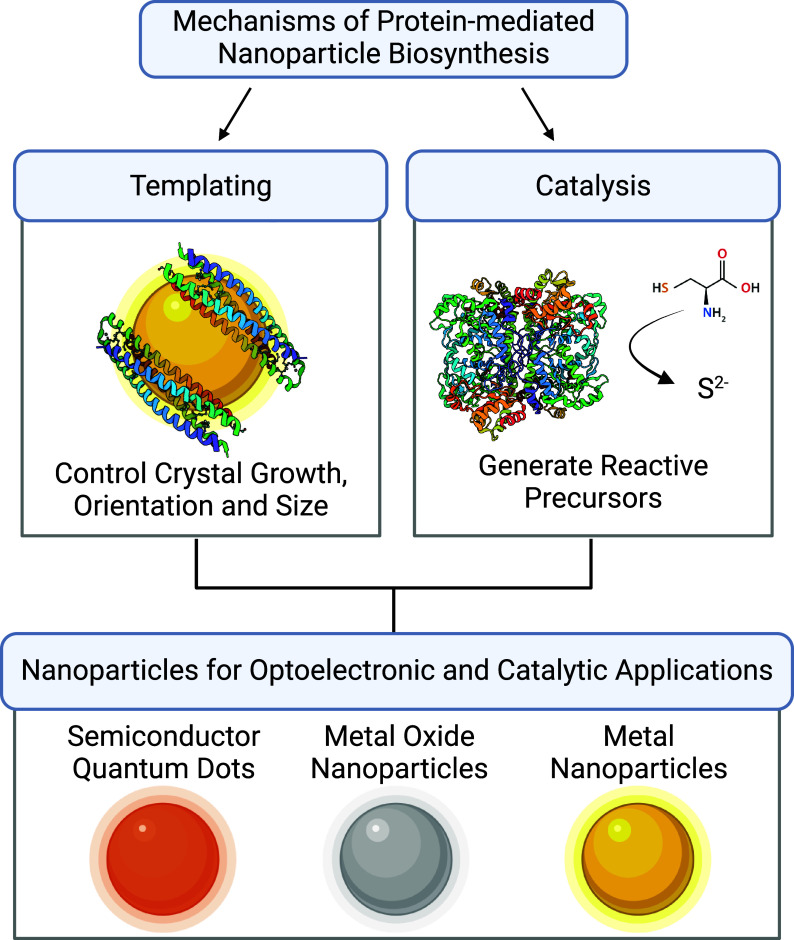
Schematic overview. For each type of functional nanoparticle, protein-mediated synthesis via templating and catalysis will be summarized.

Biomineralization pathways can be categorized into two main groups: proteins that template mineralization of a specific crystal structure or architecture, and enzymes that catalyze a reaction to induce mineralization (Figure 1). In the first type of biomineralization, proteins or peptides template crystallization to produce materials, often with exquisite hierarchical structures.^31^ For example, single cell diatoms have exquisitely complex silica skeletal structures. These structures are formed by three types of proteins: frustulins, HEPs, and silaffins. Each protein has specific repeats of amino acids bearing functional groups that help guide mineralization, such as sulfur rich regions, highly charged regions, or a large number of polar groups.^1,32,33^ The second type of biomineralization uses an enzyme to drive mineralization via reactions. One example of this method is found in *Stenotrophomonus maltophilia*, which produce the enzyme cystathionine γ-lyase (CSE) in response to heavy metal exposure. CSE produces H_2_S from cysteine, resulting in the precipitation of metal sulfide crystals that are less toxic to the bacteria.^34^ A few organisms employ both templating and catalysis mechanisms by coupling multiple types of proteins together spatiotemporally in the biochemical pathway.^35^

The main advantages of using biomineralization for materials synthesis are the aqueous, ambient conditions of synthesis, and the use of proteins to synthesize materials from the bottom-up; that is, the crystal structure is built during the assembly of individual atoms into the material. The use of bottom-up synthesis allows organisms to produce highly ordered or patterned structures at low temperatures and without additional processing steps. Biomineralization thus stands in stark contrast to traditional methods that often use top-down approaches to assemble the material postsynthesis. In these cases, achieving highly ordered structures typically requires specialized equipment and high pressure/temperature conditions to achieve desired crystallization and assembly.^36,37^

While biomineralization offers an alternative route for the synthesis of a variety of complex functional materials sustainably at scale, the final resulting material often show low performance when used in technical applications.^38,39^ The loss of performance is a result of using pre-existing pathways evolved in nature. Such pathways do not aim to produce materials for use in energy applications, such as solar cells or photocatalysts. By understanding the relationship between the proteins evolved by nature and the final product, we can begin to engineer and optimize these systems in the context of functional material synthesis. Such work has only been considered for the past 20 years.^40,41^ In comparison, traditional synthesis techniques have been well developed over 50+ years, boast a wide range of methods, and have been optimized to afford high degree of control. In order for biomineralization to be competitive with traditional manufacturing techniques, more attention needs to be given to the underlying biochemical pathways for subsequent optimization of the synthesis processes while maintaining desirable synthesis conditions.

To this end, we review the current state of the field of biomineralization for the synthesis of functional nanocrystals, focusing on the relationship between the biochemical pathway used for synthesis and the final material quality. We will overview the biomineralization of specific types of functional nanocrystals based on their end-use application, giving examples of biomineralization pathways that have been used to synthesize these materials. Instead of focusing on the numerous proof-of-concept studies found in various organisms, we aim to distill biomineralization down to several types of general biochemical pathways observed in nature, and demonstrate the effect biochemical synthesis has on the final properties of the functional material. We also highlight examples where the biochemical pathway has been modified synthetically to improve the final material properties or add control to the synthesis. Finally, we compare the resultant properties of biosynthesized materials to their traditionally synthesized counterparts and elucidate needed improvements that will allow biomineralized materials to match or surpass the performance of functional materials synthesized using well-studied traditional methodologies.

This review does not aim to show all existing examples of biomineralization but focuses primarily on biomineralization that can be used to produce functional materials for nonbiological applications, such as in photovoltaics, catalysis, or optoelectronics (Figure 1). There are several excellent reviews and books on biomineralization generally,^1,42,43^*in vivo* biomineralization for green synthesis,^26^ the exploitation of biomineralization for medical or biological applications,^26^ and the use of proteins and peptides for biomimetic synthesis of inorganic materials.^18^ Here, we focus on the relationship between material quality and the biochemical synthesis method to elucidate future developments for improving nanocrystal quality and controlling resultant materials properties of functional nanoparticles, such as size, crystallinity, and shape. Such relationships can be used to tailor protein-mediated synthesis of optoelectronic and catalytic materials that are competitive with traditional manufacturing processes.

## General Theory of Nanoparticle Nucleation and Growth

Understanding the protein-mediated biomineralization of functional nanoparticles first requires a fundamental knowledge of nucleation and growth theory. Here, we briefly outline the theoretical aspects that are relevant to nanoparticles synthesized by both biomineralization and abiotic chemical synthesis. There are several excellent reviews that describe nucleation and growth theory in complete detail.^45^

The theory of nanocrystal formation as proposed by La Mer et al. follows the general mechanism of hydrosol formation. Rooted in the Gibbs–Thomson equation that dictates nanocrystals of sufficiently small size have increased solubility in solution, nanoparticle crystallization occurs in three main stages: monomer accumulation, nucleation, and growth.^46^ Monomer accumulation refers to the stage prior to solid precipitation where the concentration of precursors is less than the supersaturation condition required for precipitation of the final material. In the case of metal chalcogenide quantum dot synthesis, this step implicitly contains the reaction of the metal and chalcogenide precursor, i.e. Cd and S, to form a soluble species of CdS, also known as a sol. Nucleation occurs when monomer accumulation of the metal chalcogenide sol surpasses the supersaturation condition, thus resulting in precipitation of a solid phase nanocrystal. Ligands also play an important role in determining the supersaturation concentration by influencing the solubility of the monomer precursors and the CdS sols. At the supersaturation point, particles will nucleate at a critical diameter that is a function of the sol concentration in solution.

Subsequent particle growth then occurs in two regimes classified by the dispersity of average nanocrystal size in solution: size focusing or size broadening. The growth regime is dictated by the relationship between the average diameter of particles in solution and the critical diameter which is based on the available monomer concentration. The critical diameter varies inversely with the concentrations of monomers in solution and will remain small when the concentration of monomers remains high. A small critical diameter relative to the average nanoparticle size results in size focusing behavior. As the monomers are consumed, the free monomer concentration drops, resulting in a larger critical diameter that will drive growth into the size broadening regime.

The above proposed classic nucleation and growth theory often works well when describing the formation of metal sulfide quantum dots or metal nanoparticles. However, recent work by Gebauer and Cölfen have proposed an updated, more nuanced description of nucleation and growth theory that addresses the formation of prenucleation clusters that likely form prior to crystallization in most protein-driven biomineralization process, especially in the case of metal oxides.^47,48^ Prenucleation clusters have often been observed in CaCO_3_, silica, and even gold metal systems, which are common biomineralization materials.^49,50^ Metal oxides are more commonly associated with prenucleation clusters, as the precursors are often transition metals which easily complex with ligands and form hydrated species in water, such as solgels.^51^ In some systems, such as iron oxides, prenucleation clusters are not necessarily considered to form, but instead extended Fe–O chains form which eventually aggregate into crystals.^52,53^ Prenucleation clusters have also been observed to form in amino acid systems, and proteins often crystallize or associate with each other especially at high concentrations.^54^ Thus, although classic nucleation and growth theory may be applicable for some systems and gives a general framework for understanding nanoparticle precipitation in solution, a more detailed picture considers the formation of soluble prenucleation clusters and also the potential for liquid–liquid phase separation of low and high precursor concentrations in solution.

An additional mechanism of growth that often occurs in biomineralization is oriented attachment. In this type of growth, pre-existing nanocrystals join together, often at faceted surfaces with the same crystallographic axis.^55^ Oriented attachment can result in a single crystal or cause a planar-types defect such as twinning. Oriented attachment has been observed in aqueous quantum dot synthesis,^56−58^ in nature, and in the formation of metal oxides. Oriented attachment also allows the formation of higher order nanostructures such as honeycombs, nanowires, and 2D sheets.^59^

## Functional Materials Produced using Biomineralization

This review focuses on three main types of functional materials currently produced using biomineralization that are desirable for use in optoelectronic and catalytic applications: semiconductor quantum dots, metal oxide nanoparticles, and catalytically active metal nanoparticles. In each section, we first overview the traditional chemical synthesis for each material and highlight the material’s desirable functional properties. We then summarize the generalized biomineralization approaches used to make the corresponding material. In some cases, the biomineralization mechanism (e.g., templating vs catalysis) directly correlates with the resultant material quality and final properties. Relating the biomineralization mechanism to material quality relates to the performance of biomineralized nanoparticles in the final application.

### 1 Biomineralization of Semiconductor Quantum Dots

Semiconductor quantum dots are a highly desirable functional material for display and energy applications due to their tunable optoelectronic properties as a function of size.^60^ The size of each individual nanoparticle results in quantum confinement of the atomic orbitals, which modifies the energy band.^61^ Thus, controlling particle size yields control over the energy gap and subsequent optical properties.^62^ Control over optical properties is highly desirable in optoelectronic applications that require the production of bright red/green light at discrete points, such as LEDs in TV screens.^63^ QDs are also used in bioimaging applications as fluorophores, and have found use as light harvesters in solar cells.^64−66^

#### 1A Chemical Synthesis of Semiconductor Nanocrystals and Desired Properties

To achieve uniform size control required for optoelectronic applications, quantum dots synthesis typically occurs via well-established chemical methods that produce highly monodisperse populations of nanocrystals.^67^ Generally, such quantum dot synthesis methods can be understood as controlled nanocrystal precipitation. Two reactive precursors are solvated in solution, often by a coordinating ligand or solvent. Once each chemical is combined, they react to form a semiconductor species. For example, Cd and Se form CdSe stabilized by one or more of the coordinating solvents, such as trioctyle-phosphine-oxide (TOPO). In such synthesis, precipitation occurs once these crystal monomers reach a specific supersaturation condition in solution. This supersaturation condition depends on the concentration of species, but is also impacted by any coordinating ligands. Once the semiconductor particles have precipitated, subsequent growth continues while the solution is heated for incubations times ranging from minutes to hours. The initial nucleation and subsequent precipitation event are critical for achieving a specific size and the mechanism underlying these two processes greatly affects the resulting nanocrystal population.

The most commonly used method for traditional semiconductor quantum dot synthesis is known as hot-injection.^62^ In this method, the above nucleation and growth stages are controlled by a rapid injection step. To begin, a solution of organic solvent, coordinating molecules, and stabilized chalcogenide precursors are heated to 150–200 °C, often under an inert atmosphere. Next, a solution of metal precursors is rapidly injected into the solution, resulting in a burst nucleation of metal chalcogenide nanoparticles. Following injection, the temperature of the solution is lowered, often to 100–150 °C, followed by a growth phase that results in QDs of the desired size.^62^ Alternative methods often add both precursors at low or room temperature followed by heating, sometimes at high pressures, to drive nanocrystal precipitation and growth.^68^

The chemical synthesis method directly affects the final quality of quantum dots in solution. The advantage of direct injection synthesis is that the two primary steps of nanocrystal synthesis are separated; nucleation occurs almost instantaneously with an immediate consumption of all monomer species, followed by controlled growth via Ostwald Ripening.^69^ This results in control of the overall particle size for the population of nanoparticles in solution using time and temperature. However, a low concentration of monomers results in a large critical radius of nanoparticles, leading to size broadening growth behavior and thus large dispersity in nanoparticle size. For example, CdS nanoparticles with an average diameter of 2–4 nm would be considered to have a broad size distribution if the standard deviation was greater than 0.5 nm.^46^ Variations in coordinating ligands, solvents, and metal precursors can be tuned to control the nanocrystal synthesis, but postprocessing of the nanoparticle solutions is often required to achieve monodisperse populations of semiconductor quantum dots.^70,71^

To gauge the overall nanocrystal size and dispersion in solution, several techniques are used. Typically, absorbance measured by UV–vis is critical for determining the average energy of the particles, and thus size. Fluorescence measurements are also used to determine the emission energy and Stokes shift, which helps to determine the type of emission mechanism occurring within the QD. The size distribution can be estimated from the broadening of the fluorescence, quantified by the full width half max (FWHM) of the fluorescence spectra. A more accurate size measurement requires TEM images of individual nanoparticles. Once nanocrystal size confirmed by TEM is correlated to the relevant absorbance peak, the latter can be used as a metric to determine nanocrystal size in solution during growth.

Another important parameter of semiconductor quantum dots are their fluorescent quantum yields (QY), which specify the amount of energy converted into fluorescent emission. QY is defined as the number of photons emitted in fluorescence over the number of photons absorbed.^72^ Measuring QY allows researchers to assess whether energy is lost within the quantum dot as a result of defects within the crystal or on the crystal surface. For example, a QD with no defects will have a QY of 100%; that is, all the energy is converted into fluorescence. If some of the energy is lost to defect through nonradiative pathways, the QY will be less. Thus, QY is a convenient measure of nanocrystal quality. However, QY is often affected by the capping ligand, so care should be taken when comparing QDs of similar materials with different capping ligands.

#### 1B Biosynthesis of Semiconductor Quantum Dots

As discussed in the previous section, abiotic quantum dot synthesis methods utilize inert atmosphere, high pressure/temperature, toxic solvents, and expensive capping ligands. These methods also require batch processes and extensive downstream purification including centrifugation and separation. While effective, these chemical syntheses are very expensive at large scale, and thus limited in commercial use. To make quantum dots viable for commercial applications, biomineralization offers a method that is continuous and sustainable using environmentally friendly solvents and techniques.

When comparing abiotic synthesis to biomineralization, there are a few major differences from a mechanistic perspective. In contrast to chemical syntheses where nucleation and growth are distinct, these events can typically not be separated using biomineralization. Separation is often not possible because the protein generates required precursors needed to drive the reaction at a slow, consistent rate. In the case of templating, low temperatures may result in slow reaction rates as opposed to a rapid burst of nucleation, or may cause the formation of prenucleation clusters discussed more in depth below.^48^ Other major differences include growth temperature (ambient), solvent (water), and capping ligand (proteins). In biomineralization, nanoparticle size and crystallinity are often constrained because of the low temperature that limits the energy required for growth via Ostwald ripening or Oriented Attachment.^49^ However, alternative methods for controlling particle size can be employed. For example, biomineralization often uses biochemical pathways that continuously produce reactive chalcogenide precursors, which limits the reaction to form quantum dots. Growth is controlled by slowly introducing monomers into the solution throughout the growth period, resulting in size focusing growth behavior. Nanocrystal size can also be dictated by the protein or biomolecule used as a capping ligand. The side group of each protein or biomolecule yields different binding strength, resulting in a specific nanocrystals size for each material-protein combination.

Generally, one of two types of biomineralization approaches are used for quantum dot synthesis: templating an artificially induced chemical reaction, or catalysis of reactive precursors. Only one approach is used at a time although in some cases both templating and catalysis are achieved by coupling multiple biochemical pathways within an organism. Most examples of quantum dot biomineralization are reported on metal chalcogenide systems, such as metal sulfides or selenides, likely because metal chalcogenides readily crystallize at low temperatures,^73^ are easily stabilized by several functional groups of amino acids, and have biologically relevant precursors such as cysteine and selenocysteine. Biological chalcogenide precursors are often easily converted to sulfur and selenium using pre-existing biochemical pathways.^74^

Below is a review of quantum dot biomineralization with a focus on specific proteins and biochemical pathways. We summarize and categorize current QD biomineralization work into two general approaches: 1) templating nanocrystal formation and 2) catalyzing the biomineralization reaction.

##### 1B.1 Templated Biomineralization of Semiconductor Nanocrystals

Templated biomineralization uses proteins and biomolecules to template and stabilize semiconductor quantum dots. Generally, the mechanism relies on metal–ligand binding interactions between amino acid residues on the protein and the material surface. Overall, the mechanisms are not well-defined as very few studies on metal surface-ligand binding have been performed in contrast to the numerous studies on individual metal ion-protein binding such as in metalloproteins.^75,76^ However, one shared mechanistic feature is that the final nanocrystal size is controlled by metal chelation to the surface of the quantum dot, which blocks growth sterically. Metal chelation is achieved by functional groups of amino acids on the protein, such as thiols (C), imidazolium (H), or positive/negative charge (K, R, E, or D). The binding strength of the capping ligand together with the concentration of the precursors in solution dictates a final nanocrystal size by affecting the equilibrium supersaturation condition. Growth of nanocrystals is possible by changing the equilibrium condition via continuous or titrated introduction of the chalcogenide precursors. Here, we outline several classes of proteins and biomolecules used for templating semiconductor quantum dot synthesis and demonstrate their control over subsequent nanoparticle size, shape, and morphology. While many papers cite proteins generally as stabilizers of nanocrystals, we here focus on specific proteins that have been identified to stabilize or direct growth and examine the specific aspects of the proteins that have led to controlled growth. The reviewed studies on templated quantum dots biomineralization are summarized in Table 1.

**Table 1:** Summary of Protein-Mediated Semiconductor Quantum Synthesis via Templating^a^

Semiconductor Quantum Dots				
Templating				
Protein	Nanomaterial	Size	QY	Reference
Bovine serum albumin (BSA)	CdS	4 nm	Low	(11)
	FeS	3.0 nm	Low	(78)
	Ag_2_Te	3 nm	2.3	(79, 80)
Bovine Serum Albumin (BSA) + thioacetamide (TAA)	PbS	15, 25, and 35 nm	NR	(81)
	HgS	20–40 nm	NR	(12)
	Ag_2_S nanorods	30 nm by 90–240 nm	NR	(82)
	Bi_2_S_3_	<10 nm	NR	(83)
Phytochelatins	CdS	1.6–2 nm	NR	(85, 86)
	CdSe, CdTe, and ZnSe	4.99 ± 0.69, 5.83 ± 1.60, and 3.95 ± 1.12 nm	NR	(89)
Ferritin cages	CdS	4.2 ± 0.4 nm	NR	(95)
	AuS	6.1 ± 0.4 nm	NR	(94)
	ZnSe	7 nm	NR	(91)
	CdSe	7.1 nm	NR	(92)
Designed peptides	CdS and ZnS	4 and 5 nm	NR	(96)
	CdS/ZnS nanorods	4 nm by 2 nm	NR	(41)
	CdSe/ZnS	5–20 nm	NR	(97)

a This is representative but not inclusive of all examples referenced in text.

##### 1B.1a BSA Templating

**Figure 2:**
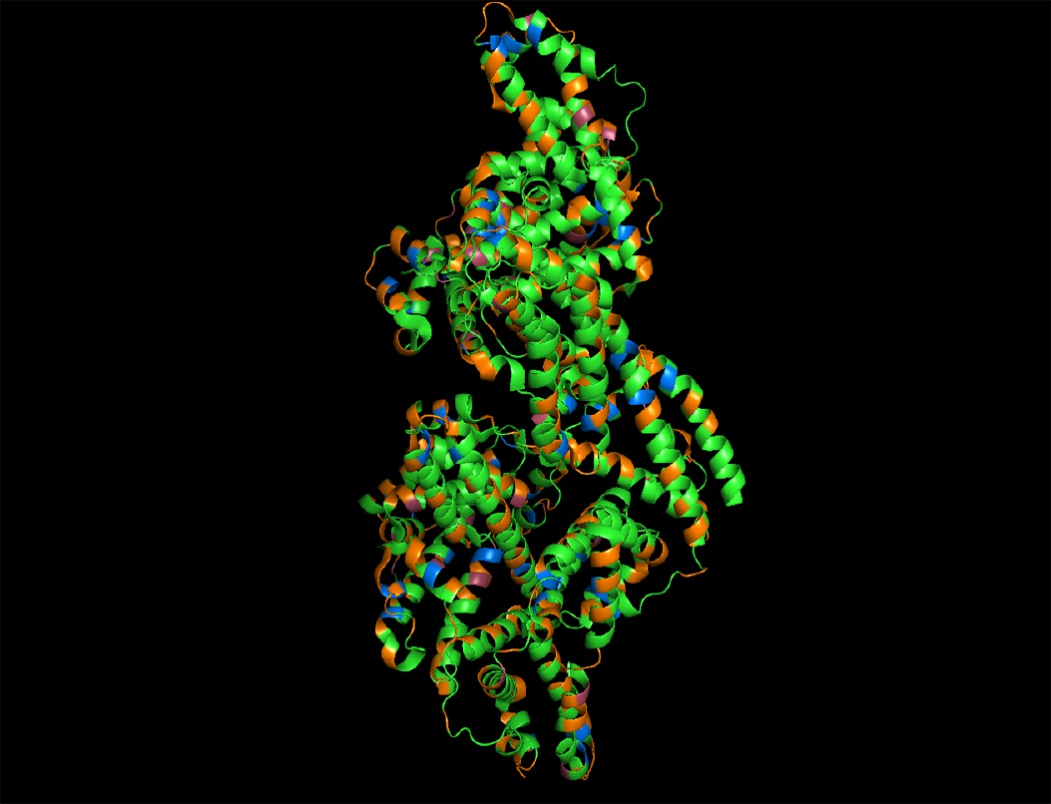
Protein structure of BSA (PDB 4F5S) with potential metal binding amino acids highlighted: Histidines are fuchsia, cysteines are blue, and charged resides are orange. Figure made with The PyMOL Molecular Graphics System, Version 2.5.2 Schrödinger, LLC.

One of the most commonly used proteins to template nanoparticle synthesis is bovine serum albumin (BSA). BSA is readily obtained as a byproduct of the cattle industry and is available inexpensively through common scientific retailers. BSA has many functional groups that are capable of binding metal, including 17 histidines (2% overall), 35 cysteines (6% overall), and numerous charged amino acids (31% overall), highlighted in Figure 2.^77^ BSA has been used to produce semiconductor nanocrystals of several varieties that are outlined below. Because BSA only acts as a templating agent to control the final nanoparticle size, a chemically induced reaction must be used to produce the reaction leading to metal chalcogenide synthesis.

In the simplest version of the synthesis, a metal precursor and a reactive chalcogenide precursor, such as Na_2_S or NaHSe, can be added to a solution of BSA to produce metal chalcogenide QDs. This approach was used by Ghosh et al. to produce CdS and CdSe quantum dots that had optical properties consistent with quantum confinement.^11^ Particle size and fluorescence intensity could be modulated by increasing BSA concentration, modifying solution pH which subsequently alters BSA conformation, or adding a reducing agent which breaks disulfide bonds within the protein making cysteine available for capping. This same approach was also used by Yang et al. to produce FeS nanoparticles. In this case, BSA produced a confined microenvironment that also helped stabilize the FeS nanoparticles for use in theragnostic imaging.^78^ Overall, these works demonstrate that increasing the availability of metal binding groups on a protein enables better control over nanoparticle size. However, nanoparticle populations are often fairly polydisperse with low quantum yields.

In addition to sulfur and selenide chalcogenides, BSA has also been used to template the formation of Ag_2_Te QDs. In this case, reactive telluride precursors were prepared by heating tellurium powder with sodium borohydride to produce NaHTe. It should be noted that sodium borohydride is a highly toxic chemical and makes this route less environmentally friendly than other biomineralization pathways. This mixture was then combined with silver nitrate complexed with BSA. Because Ag^1+^ is complexed with BSA prior to reaction, silver availability is limited which controls the reaction, producing size-constrained Ag_2_Te nanoparticles of approximately 3 nm in diameter. From a theoretical standpoint, the chelation of Ag^1+^ modifies the supersaturation condition required for particle formation, directly influencing the critical radius, final particle size, and the type of growth (size narrowing vs size broadening). As a result, the particles had fluorescence at 1300 nm with a QY of ∼2.3% and photothermal activity when excited by an 808 nm laser pulse. The nanoparticle properties are comparable to state-of-the-art chemical routes, which also suffer from low QY (<10%). Biosynthetic routes may present a potential area for improvement using shell growth or increased particle size and crystallinity to reduce recombination.^79,80^

In addition to using BSA to control the very fast reaction of Na_2_S, NaHSe, or NaHTe with metal in solution, other biogenic routes further slow nanoparticle synthesis by using an alternative sulfur precursor that breaks down over time, such as thioacetamide (TAA). Using such precursors in addition to BSA helps to further control the nanoparticle size and growth rate. TAA has been used to produce narrow band gap semiconductor nanoparticles such as PbS and HgS capped with BSA.^12,81^ TAA also chelates the metal precursor similarly to BSA, further limiting the metal availability and thus reactivity. As a result, TAA is controlling the final nanoparticle size in two ways; the first is by modifying the supersaturation condition of the metal in solution via chelation, and the second is by slow release of sulfur which continuously introduces monomer species and thus shifts the equilibrium condition during growth. Because both BSA and TAA compete for chelation of the nanoparticles in solution, nanoparticles synthesized by this approach often grow to be much larger in size, are highly crystalline and well faceted. The effect on growth is likely related to a change in the supersaturation condition resulting from two competing templating molecules.

**Figure 3:**
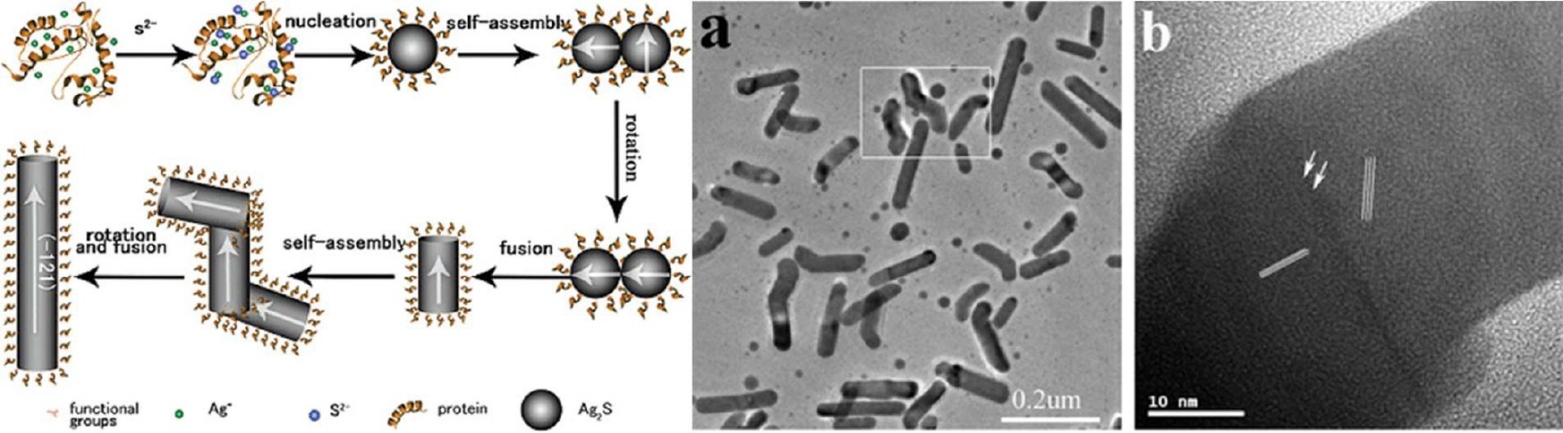
Incorporating both BSA and TAA during nanoparticle synthesis leads to control over nanoparticle morphology, such as nanorods through growth mechanisms such as oriented attachment. Adapted with permission from ref (82). Copyright 2008 American Chemical Society.

The unique dual role of TAA in BSA templated nanoparticle synthesis also allows the formation of alternative structures beyond nanoparticles. Yang et al. demonstrated the growth of Ag_2_S nanoparticles and nanorods, where nanorods formed with longer growth time.^82^ Here, the nanoparticles nucleate and initially grow following typically nucleation and growth theory. Once all the monomers are consumed and particles formed, nanorod growth in enabled by a process known as oriented attachment (Figure 3). Here, specific crystal faces align and fuse to create rod-like structures. The hypothesized growth method may involve BSA or TAA binding specifically to one facet, allowing oriented attachment to occur on specific crystal faces. However, such a mechanism is difficult to prove. Regardless, this work demonstrates that using a protein with specific chelating ability may enable the creation of higher order nanostructures.

Another method for introducing sulfur into a BSA-templated nanoparticle growth solution is using the decomposition of sulfur containing amino acids. One example of this was performed by Wang et al., who produced sub 10 nm Bi_2_S_3_ nanoparticles.^83^ In this case, bismuth precursors and BSA were mixed to create a chelated Bi-BSA species. Then, the pH was adjusted to 12, causing the decomposition of cysteine on BSA into S species that then controllably reacted with the locally bound Bi. This method is advantageous because the sulfur is released in specific regions in close proximity to Bi. Also, the S is released slowly as cysteine decomposes at high pH. This results in better particle size control compared to other BSA templating methods.

BSA has been shown to form a hydrogel when stabilizing metal chalcogenide nanoparticles. With high levels of BSA, electrostatic coordination between protein molecules, along with hydrogen bonding, condense the mixture to a high viscosity liquid, or hydrogel. Within this BSA hydrogel, there is still protein-metal coordination that ultimately stabilizes the particles.^84^ This approach demonstrates the unique capabilities protein-based synthesis techniques offer for creating quantum dot-hybrid materials. However, the particle size depends entirely on the hydrogel properties such as pore size, and the facile formation of a hydrogel indicates that similar studies on BSA-stabilized QDs formed in aqueous solutions are actually clustered into protein matrices. While this clustering is ideal for hydrogel formation, such properties may limit biosynthetic quantum dots from being used in applications unless the BSA clustering can be leveraged for a specific purpose, such as patterning or locating quantum dots in a specific place.

##### 1B.1b Phytochelatins

Another class of proteins that are shown to produce quantum dot materials are phytochelatins, which are naturally occurring peptides produced by many species including plants and yeast.^85^ Phytochelatins (PC_n_s) typically possess the amino acid sequence (g-Glu-Cys)_n_-Gly where the number of repeating units, n, varies between 2 and 11.^86^ This high concentration of cysteine enables stabilization of ultrasmall CdS nanoparticles with optical properties consistent with that of quantum dots. The ability of phytochelatins to stabilize CdS quantum dots was initially observed in marine phytoplanktonic algae (*Phaeodactylum tricornutum*) exposed to Cd, where phytochelatins of varying lengths (n = 2–6) were found to be associated with quantum confined CdS crystallites.^87^ Following this work, Chen et al. overexpressed PC_n_s in *E. coli* and showed that controlling the length of the PC modulates the final size of the CdS nanocrystal. This work demonstrated that the presence of more cysteine in the peptide results in smaller nanoparticles, inferring the presence of more binding groups slows or stops nanocrystal growth at a defined size.^86^ Additional work by Chekmeneva et al. demonstrated via isothermal calorimetry (ITC) that PC_4_ binds Cd at two binding sites as compared to one in PC_3_, consistent with the observed smaller CdS nanoparticle size with longer PC.^88^ Compared to using monomeric cysteine or glutathione (GSH), PC_n_ have the advantage of strongly controlling particle size and improving stability of the nanocrystals over longer periods of time, which are important factors when applying biogenic nanoparticles in applications.

In work by Park et al., both phytochelatins and metallothioneins were observed to stabilize quantum confined nanoparticles of CdSe, CdZn, CdTe, and ZnSe. Samples of CdSe nanoparticles were able to fluorescence, and the average size of each type of nanoparticle was measured using TEM. In this case, the quantum dots were observed in *E. coli* cells recombinantly expressing phytochelatins and metallothioneins, and therefore the cell may also be performing a catalytic reduction step to produce reactive Se, Te, and S. Although not addressed in the work, these pathways will be discussed in the following section.^89^

##### 1B.1c Cage Proteins

**Figure 4:**
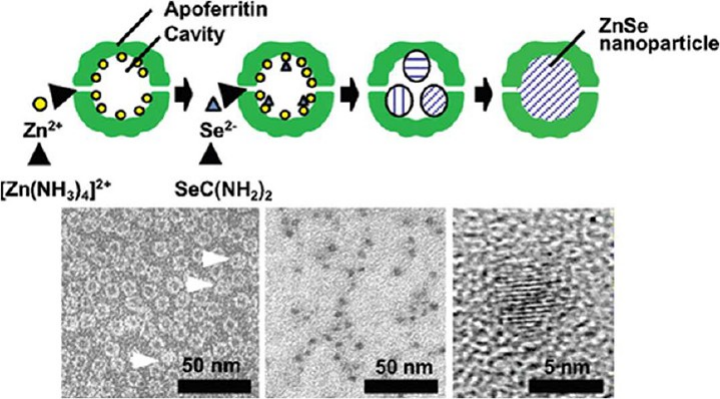
Schematic of ZnSe nanoparticle formation using an apoferritin cage. Nanoparticles are controlled in size to ∼4 nm as confirmed with TEM. The first TEM panel shows both nanoparticle and protein using 1% aurothioglucose staining. Reproduced with permission from ref (91). Copyright 2005 American Chemical Society.

Another class of proteins that has been used to produce quantum confined nanocrystals are ferritin proteins. These proteins form protein “cages” and are typically used to shuttle iron around biological systems, with iron ions entering the cavity of the protein through hydrophilic channels where they can be stored for use. Ferritin was first shown to produce CdS nanocrystals by the group of Steven Mann, who demonstrated the particles had blue-shifted absorbance spectra consistent with quantum confinement.^10^ Following this work, ferritin and apoferrtin were used to produce AuS, CdSe, and ZnSe nanocrystals, as shown in Figure 4.^90−94^ These studies did not extensively characterize the particle size and potential optical properties but demonstrated that many types of metal chalcogenides could be produced with this method.

Several groups have also investigated the ability of specific ferritin proteins to tune the resultant properties of the nanocrystal system. A recombinant apoferritin (FerA-dcys) was shown to produce CdSe at a higher yield, attributed to the presence of two additional cysteines on the protein.^92^ The first well characterized examples of CdSe synthesis with size control were performed by Iwahori et al., who showed larger, 7.1 nm CdS particles could be produced using apoferritin from horse spleen (HsAFr), which assembles to form a larger cavity. More recently, Iwahori et al. produced CdS nanocrystals inside the cavity of Dps proteins from *Listeria innocua* (LiDps).^95^ This protein is composed of 12 identical protein subunits that assemble into a cage with a cavity of 4.5 nm. Synthesis is initiated by introducing Cd and thioacetic acid (TAA) with ammonia to a protein solution, which catalyzes the breakdown of TAA into reactive sulfur to produce CdS nanocrystals of approximately 4.2 nm in size. These CdS nanocrystals also had photoluminescence consistent with that of quantum confined nanocrystals. This work demonstrates the ability of self-assembling proteins to strongly confine particle size to a desired radius. By tuning such protein cages to specific sizes, highly uniform quantum dots of specific size could be easily assembled at room temperature as needed.

##### 1B.1d Designed Peptides

Thus far, the templating proteins discussed have been naturally occurring proteins that coincidentally template the formation of QDs, aside from their native applications. Many groups are now investigating the use of non-natural peptides that are designed specifically for metal binding of nanomaterials. The leader in this area is Dr. Angela Belcher’s group, who have identified many metal binding peptides using the M13 phage display system for common semiconductor materials such as CdS or ZnS. Initial work by Mao et al. identified specific amino acid motifs that correspond to strong binding of metal chalcogenide surfaces.^41^ Importantly, both the sequence of amino acids and the structure of peptide (linear or constrained) influenced the morphology and crystal phase of the CdS or ZnS formed in solution. This work also points to amino acids that likely play strong roles in confining nanocrystals, including sulfur rich amino acids (C, M), positively charged amino acids, and H. Notably, there was at least one H present in every sequence found to bind metal strongly. Interestingly, negatively charged amino acids were observed very infrequently, and do not seem to be favorable for binding to metal chalcogenide surfaces.

**Figure 5:**
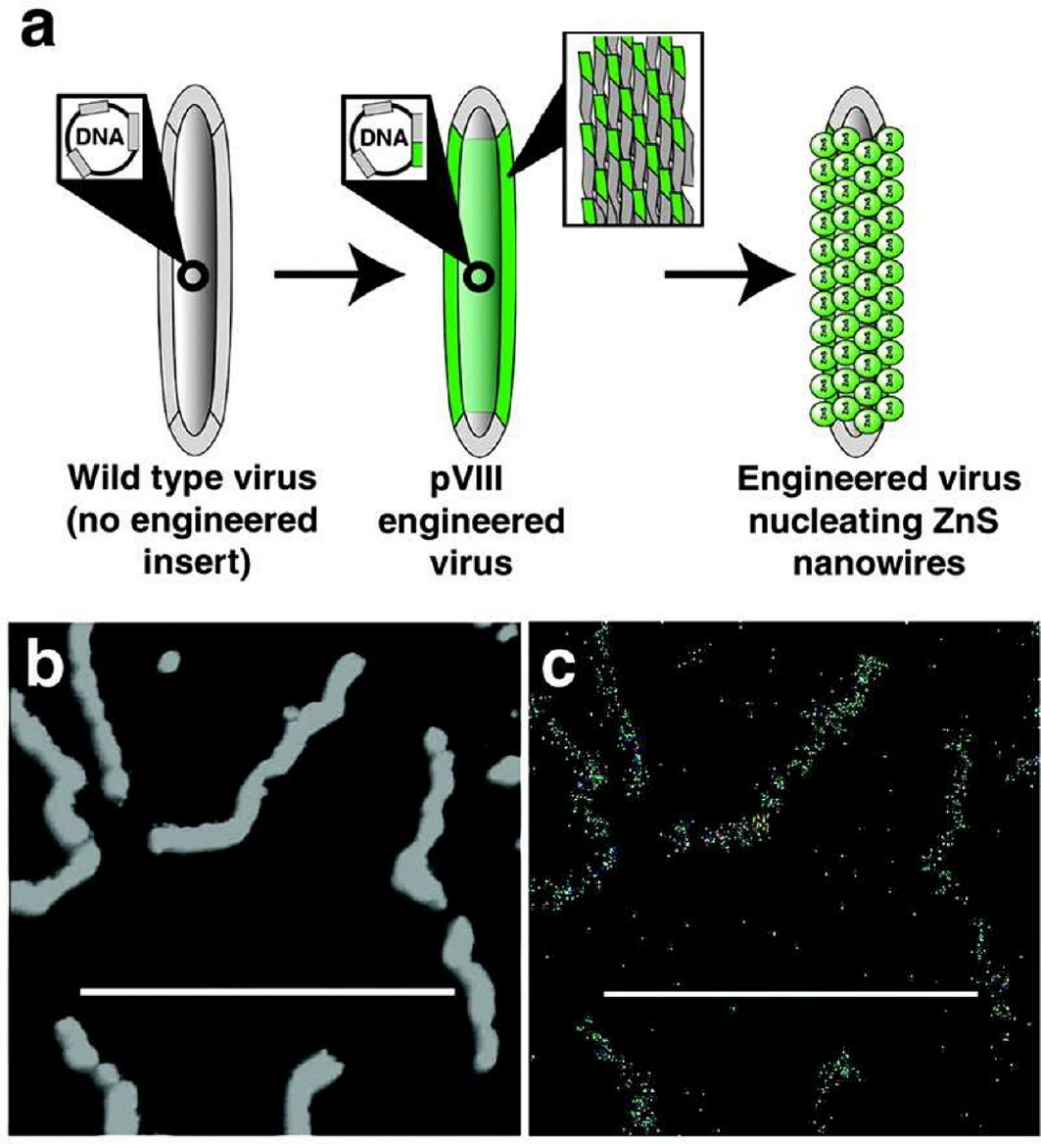
a) Schematic of ZnS nanowires synthesized using A7–pVIII-engineered viruses engineered specifically for biomineralization of ZnS. b) TEM imagining of ZnS nanowires coupled with c) EDS mapping to confirm the presence of S within the nanowires. Reproduced from ref (96). Copyright 2003 National Academy of Sciences, U.S.A.

Use of M13 not only enabled the discovery of peptides with binding affinity for specific semiconductor surfaces and orientations, but was also used to template the formation of metal chalcogenide nanowires as shown in Figure 5. M13 phages self-assemble into linear, wire-like structures, so the introduction of the reactive precursors Cd and Na_2_S, which react to form CdS along the peptides displayed by the phages, can be guided to form extended CdS nanowire structures. This same method was also used to produce highly crystalline ZnS nanowires and CdS/ZnS heterostructured systems.^96^

Following these seminal works, Singh et al. demonstrated that these peptides could be joined into a bifunctional peptide capable of mineralizing CdSe/ZnS core/shell nanoparticles.^97^ Taking sequences from Belcher’s work, Singh created a dually functional peptide by connecting two separate CdS and ZnS binding regions into a single peptide. A rigid proline linker was used to enable the peptide structure of each region to remain intact. The Cd binding region was of a confined structure due to the cysteine linkage, and the Zn region was linear. This peptide clearly demonstrated the formation of CdS/ZnS core/shell structures, as observed using HAADF-STEM imaging. However, the optical properties were relatively unaffected due to the thickness of the ZnS shell. This work shows that understanding and incorporating functional amino acid sequences and structures together can enable highly complex nanomaterials to be synthesized for use in nonbiological applications.

##### 1B.2 Biomineralization to Catalyze the Growth of Semiconductor Nanocrystals

The first demonstrations of quantum dot biomineralization were found in organisms that were capable of surviving toxic concentrations of heavy metals. Thus, most biomineralization approaches to quantum dot synthesis exploit the response of an organism to toxic metals or chalcogenides to produce metal chalcogenide quantum dots. In most cases, these organisms simply upregulate sulfur reducing pathways to make reactive sulfur that can then react with the heavy metal, reducing its toxicity and enabling quick export from the cell or organism. As a result, the majority of quantum dots fabricated using biomineralization are metal sulfides. Other chalcogenides such as selenium and tellurium can also occur often in nature, but are highly toxic relative to sulfur. Due to their chemical similarity, they can typically be reduced by pre-existing sulfur reducing pathways.

While the main catalytic step to produce nanoparticles is the spontaneous reaction of M + Chalcogenide → MC, the more important and rate limiting step is often the biochemical pathway(s) that produce an easily reactive sulfur or selenium species. Recent work has focused on uncovering the enzymes and biosynthetic pathways that produce sulfur and selenium in metal chalcogenide producing organisms. Here, we summarize the main pathways based on each chalcogenide, organized by the relevant enzyme class to show common trends across organisms capable of metal chalcogenide production. Except in a few cases, most quantum dot biomineralization occurs within organisms. When available, we highlight *in vitro* biomineralization using a specific enzyme to motivate future work on identifying and isolating enzymes from organisms that can be employed for purely protein-mediated synthesis. A summary of this section is provided in Table 2.

**Table 2:** Summary of Protein-Mediated Semiconductor Quantum Synthesis via Catalysis^a^

Semiconductor Quantum Dots				
Catalysis				
Protein	Nanomaterial	Size	QY	Reference
Sulfite reductase	CdS	3–5 nm	NR	(99)
Cysteine desulfhydrase	CdS	8 nm	NR	(102)
	CdS	2.31 ± 0.51 to 2.59 ± 0.78 nm	7.8–21%	(104)
	CdAgS	7.20 nm	31.36%	(103)
Cystathionine-γlyase	CdS	2.75 ± 0.68 to 3.36 ± 0.98 nm	3 to 12%	(34, 109)
	PbS and PbS/CdS	4 nm	16 to 45%	(39)
	CuInS_2_, CuInZnS, CuInS_2_/ZnS	2 to 2.2 nm	0.10%	(110)
	CdZnS and CdS/ZnS	2.7 ± 0.44 nm	5.21% and 7.02%	(111)
	ZnS	2.55 ± 0.48 nm	1.88%	(111)
	CdSe	2.74 ± 0.63 nm and 4.78 ± 1.16 nm	12%	(125)
*De novo* protein ConK	CdS	3 nm	0.30%	(115)
Reductases	CdS_*x*_Se_(1–*x*)_	2.0 ± 0.4 nm	5.2%	(120)
	CdSe	3.3 ± 0.2 nm	7.3%	(121)
	CdSe	3.0 ± 0.3 nm	NR	(122)
	CdSe	2.3 ± 1.3 to 3.6 ± 1.6 nm	NR	(124)
	CdTe	2.33 ± 0.59 nm	8.3%	(129)

a This is representative but not inclusive of all examples referenced in text.

##### 1B.2a Sulfur Reactions

The majority of work on metal sulfide quantum dot biomineralization uses bacteria, yeasts, or plants that reduce sulfate to a reactive form of sulfur, likely S^2–^ or HS^–^. However, several organisms have been observed to produce reactive sulfur directly from a sulfur containing biomolecule such as cysteine, glutathione, or methionine. Here, we demonstrate both approaches and categorize each specific enzyme, if elucidated.

##### 1B.2a.i Sulfite Reductase

The production of a reactive sulfur species from sulfite species was first observed in the yeast *Fusarium oxysporum*. Shown in work by Ahmed et al., *F. oxysporum* produced CdS nanoparticles from CdSO_4_ precursors over a time span of 12 days.^98^*F. oxysporum* is known to produce sulfite reductases, so this was hypothesized to be the enzyme responsible for metal sulfide growth. This hypothesis was confirmed by Ansary et al. in another work, where sulfite reductase was purified from *F. oxysporum* and used to produce CdS quantum dots *in vitro*.^99^ The reaction mixture also required NADPH to enable reduction of CdSO_4_ to a reactive sulfur species. While the nanoparticles show quantum confined absorbance spectra, the TEM analysis appears to show particles larger than the expected 3–5 nm.

##### 1B.2a.ii Cysteine Desulfhydrases

Several metabolic pathways exist in cells that are capable of breaking down the naturally occurring biomolecule l-cysteine into products such as S^2–^, pyruvate, and ammonia.^100^ The most common enzymes associated with producing H_2_S are cysteine desulfhydrases and cystathionineγ-lyases. We first discuss the used of cysteine desulfhyrases for quantum dot biomineralization.

**Figure 6:**
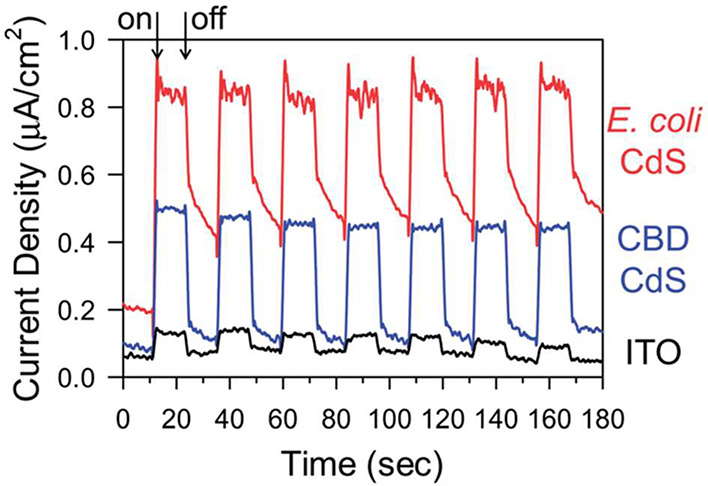
Transient photocurrent testing of bacterially synthesized CdS NPs. Reproduced from ref (102). Copyright 2016 Royal Society of Chemistry.

Many organisms produce the enzyme cysteine desulfhydrase as a response to high levels of toxic heavy metals such as cadmium. For example, Bai et al. demonstrated that *Rhodopseudomonas palustris* would produce ∼8 nm CdS crystallites when exposed to high concentrations of cadmium sulfate.^101^ Using a protein gel, four cysteine desulfhydrases were identified to be upregulated and associated with the formation of CdS nanocrystals. Marusek et al. further proved this mechanism by cloning the gene for cysteine desulfhydrase from *Treponema denticola* into *E. coli* to overexpress the protein, enabling programmed CdS growth rather than relying on a heavy metal response. In this case, l-cysteine was directly supplemented into the growth media in order to provide an overabundance of bioavailable sulfur rather than relying on an internal pool, accelerating the growth time of CdS nanoparticles. The CdS nanocrystals were extracted and applied onto conductive indium-doped tin oxide (ITO) glass and shown to produce photocurrent when exposed to light, suggesting they can be used in optoelectronic applications (Figure 6).^102^ However, in each of these preliminary studies, the CdS particles were not definitively shown to be quantum confined and their size could not be controlled.

Later work by the group of Pérez-Donoso demonstrated size control of quantum dots using *E. coli*. In this work, over production of naturally occurring cysteine desulfhydrase in *E. coli* was hypothesized to be a survival response to heavy metal exposure.^103^ Similarly to Marusek et al., l-cysteine was added to the growth media to speed up nanocrystal growth to occur over 1.5 h. Here, cadmium and silver were used to produce quantum confined CdAgS alloyed nanocrystals, demonstrated by a blue-shifted absorbance and fluorescence compared to bulk CdS and then a subsequent shift to the near-infrared region with the addition of silver.

Pérez-Donoso showed an additional strain of arctic bacteria *Pseudomonas deceptionensis* that was capable of producing quantum confined CdS quantum dots using either cysteine or methionine as a sulfur source.^104^ In the case of cysteine, cysteine desulfhydrase was assumed to produce reactive sulfur. However, when methionine was used as a sulfur source, the enzyme methionine-γ lyase was required to produce MeSH which could subsequently be broken down to produce reactive sulfur. In this work, the quantum dots were evaluated for standard quantum dot metrics such as quantum yield, and found to have 21% and 7.8% quantum yields, respectively.

##### 1B.2a.iii Cystathionine γ-Lyases

While several bacteria have been identified to produce reactive sulfur species in response to heavy metal, little work has been done to identify and independently use the enzyme for extracellular production of quantum dots.^105−107^ However, work from the groups of Steven McIntosh and Bryan Berger has identified a novel cystathionine γ-lyase (smCSE) produced by the bacteria *Stenotrophomonas maltophilia*.^34^ This enzyme was shown to be produced by *S. maltophilia* in the presence of high levels of Cd or Pb and l-cysteine, resulting in the production of size controlled CdS and PbS quantum dots. Importantly, the CdS and PbS quantum dots could be produced at a variety of sizes simply by varying growth time and could be phase transferred into the organic phase for application, if needed.^39,108^ Spangler et al. further showed for the first time the biomineralization of PbS/CdS core/shell quantum dots by removing unreacted lead acetate and introducing cadmium acetate during growth.^39^ Importantly, the growth occurred even in the absence of *S. maltophilia*, indicating that enzymes being secreted from the cells were responsible for nanocrystal growth.

Following this work, Dunleavy et al. identified the putative enzyme cystathionine γ-lyase (smCSE) via protein gel electrophoresis of purified CdS quantum dots produced by *S. maltophilia* to identify any residual protein associated with quantum dots after growth.^109^ ESI-MS revealed smCSE, which was subsequently recombinantly expressed in *E. coli* and purified. Purified enzyme smCSE was capable of producing size controlled CdS QDs in the presence of l-cysteine, demonstrating single enzyme biomineralization of CdS *in vitro*. Future work demonstrated that CSE could be used to produce CuInS_2_, CuInZnS, CuInS_2_/ZnS core–shell, CdZnS, ZnS, and CdS/ZnS core/shell quantum dots.^110−112^ The synthesized quantum dots were used in applications ranging from quantum dot sensitized solar cells to fluorescent tagging of cancer cells with little to no postprocessing steps.

CSE’s mechanism is the production of H_2_S from l-cysteine using a PLP cofactor. CSE is from a known class of enzymes that can catalyze the reduction of l-cysteine into H_2_S, and the production of H_2_S from smCSE was confirm independently using the molecule 7-Azido-4-methylcoumarin (AzMC), which converts to amino-4-methylcoumarin AMC following interaction with H_2_S producing the fluorescent AMC. This work also demonstrated that control of CdS nanocrystal size was a result of slow, continuous production of H_2_S by the enzyme, resulting in size-narrowing of the quantum dot populations during growth, improving the particle size distribution of the quantum dots.^40^ In other work, CSE was shown to be used for the reduction of graphene oxide, demonstrating the potential to biogenically produce other types of materials.^38,113,114^ This is only possible based on a deep understanding of its mechanism, and motivates studies on other currently unidentified or not well studied biomineralization enzymes from other systems.

##### 1B.2a.iv *De Novo* Proteins

**Figure 7:**
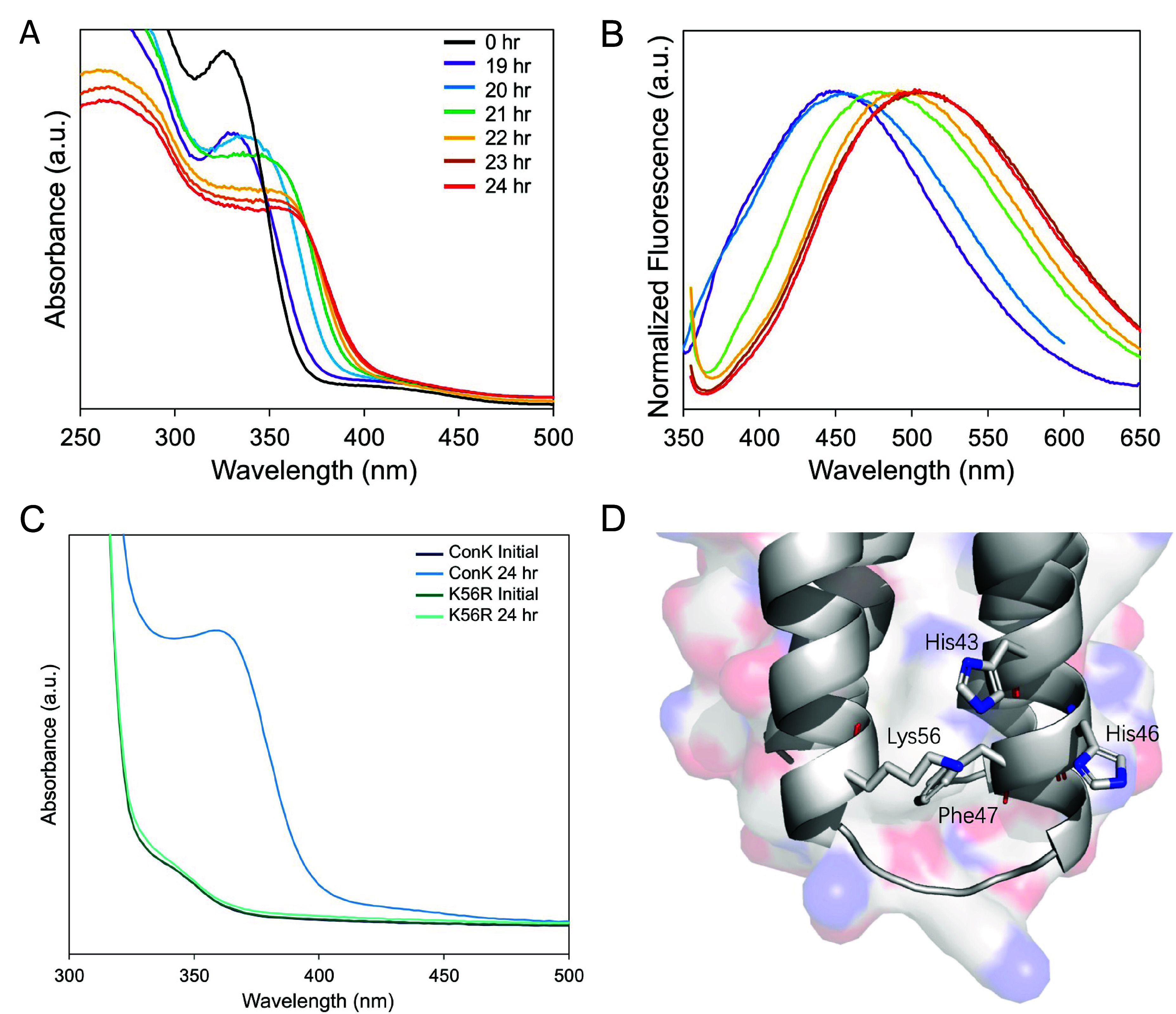
Biomineralization of CdS quantum dots by the *de novo* protein ConK. A) Absorbance and B) fluorescence spectra of CdS quantum dots grown by ConK over growth time, showing the size-dependent optical properties. C) The lack of CdS absorbance peak when the active site of ConK, Lys56, was mutated to alanine. D) Alphafold illustration of the active site of ConK. Reproduced from ref (115) under Creative Commons license for noncommercial use.

Recently, Spangler et al. identified a *de novo* protein that was capable of producing CdS quantum dots with size tunable properties.^115^*De novo* proteins stand in stark contrast to natural proteins as they are not found in nature, and instead are designed following a polar-non polar amino acid motif. Thus, *de novo* proteins are not expected to have any functionality. However, the *de novo* protein ConK was capable of binding PLP and thus was able to perform the desulfurization of l-cysteine into HS^–^, leading to the production of CdS quantum dots when Cd^2+^ was present. As shown in Figure 7, the active site of ConK was identified to be a lysine residue at position 56. Strikingly, ConK can act on both l-cysteine and d-cysteine, enabling the synthesis of chiral CdS quantum dots. The lack of stereospecificity is not found in natural proteins, and demonstrates how designing non-natural proteins can enable the synthesis of a broader range of materials with previously inaccessible functionalities.

##### 1B.2b Selenium Reactions

The preparation of metal selenide nanoparticles requires the reaction of a metal ion, such as Cd, with a reactive form of selenium, Se^2–^. In aqueous systems, Se^2–^ readily oxidizes to elemental selenium or selenium oxyanion, limiting the formation of metal selenide quantum dots *in vitro*. Thus, most previously identified protein based biomineralization approaches rely on multiple catalytic steps and enzymes that reduce selenite to a reactive selenium intermediate prior to the spontaneous reaction with a metal ion. Here, we briefly introduce enzymes that have been identified from microbes capable of reducing selenite, thus enabling the formation of metal sulfide quantum dots. In rare cases, the intermediate seleno-l-cystine has been shown as a usable precursor for metal selenide quantum dot synthesis. However, most work on the production of metal selenide revolves around systems that produce reactive precursors by reducing elemental selenium or selenium oxyanion species.

##### 1B.2b.i Reductases

Most examples of biological reduction of selenite occur in organisms responding to the high toxicity of selenium. These responses are often based off of pre-existing sulfur respiration pathways, and thus use sulfur containing biomolecules and intermediates such as glutathione.^24^ The exact route of selenite degradation is contested, mainly because the identification of intermediates is difficult within the cell. One commonly proposed route observed in yeast begins with sodium selenite, Na_2_SeO_3_, being first reduced to a bioavailable form of selenium such as selenodiglutathione or selenomethionine.^116^ This step has been proposed to be abiotic, occurring spontaneously in the cell, or through reaction with glutathione, producing selenodiglutathione (GS-Se-SG).^117^ Following this step, glutathione reductase is proposed to further reduce GS-Se-SG to GS-Se, which can then spontaneously react with Cd to produce CdSe. Several examples demonstrate upregulation of glutathione and glutathione reductase, supporting this proposed pathway for CdSe synthesis.^116,118,119^

In recent work by Tian et al., the above pathways were identified in *E. coli* grown in media with excess glucose, allowing upregulation of glutathione producing pathways and reductive enzymes.^120^ Two specific reductases were identified using ESI-MS to be thioredoxin and glutaredoxin. The more prevalent enzyme was glutaredoxin, but both are capable of reducing selenite into selenodiglutathione. The authors also showed the potential for each protein to bind Cd, enabling nucleation and growth of particles. However, the dual nature of the protein was only theoretically demonstrated in calculations, and not proven experimentally by X-ray crystallography or measured binding. In more recent work, Tian et al. took this work further by demonstrating that performing biosynthesis at the specific pH 4.5 enhanced CdSe production and fluorescent quality.^121^ Increased quantum dot fluorescence was coupled with an increase in total protein, glutathione concentration, and gene expression for glutaredoxin production. The ability to tune nanoparticle synthesis demonstrates how focusing on engineering the biosynthetic pathway and specific proteins may enable the production of other technologically relevant nanoparticles.

**Figure 8:**
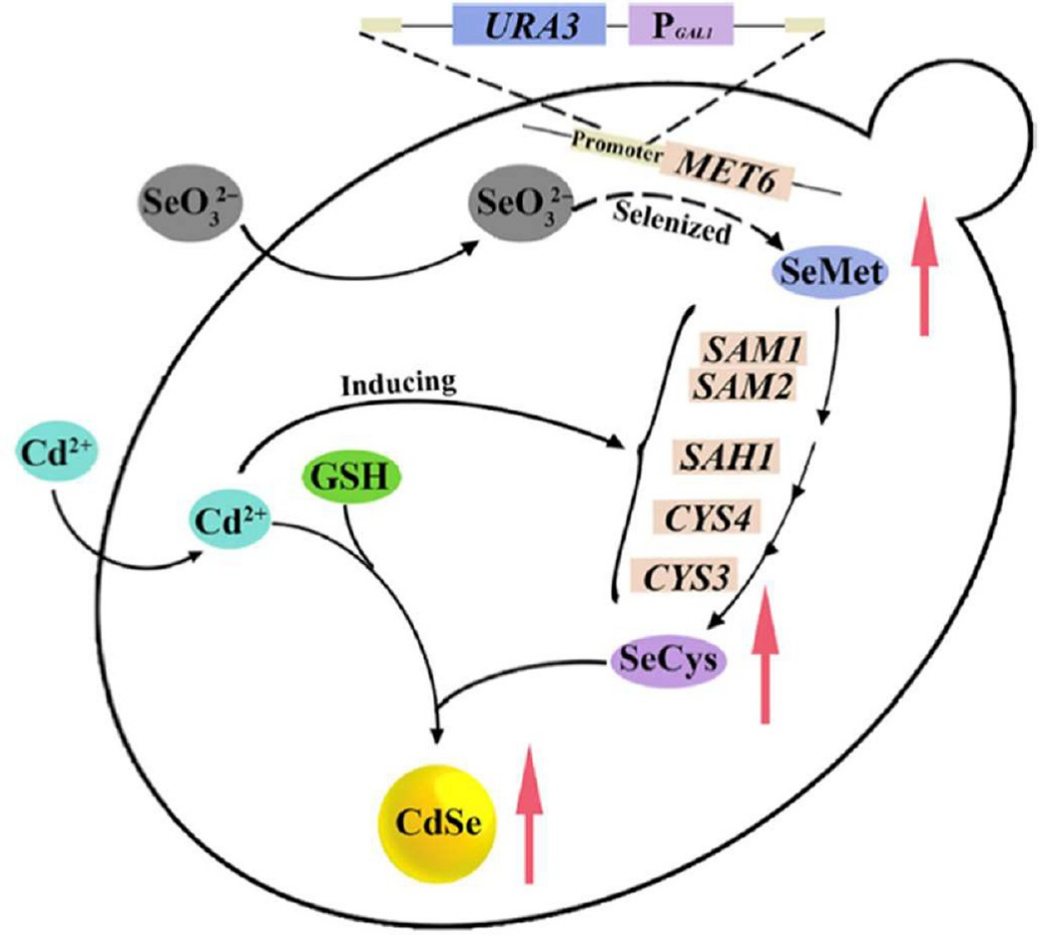
Multiple pathways for selenite reduction and the coupling of Cd^2+^ shown to produce CdSe QDs. Importantly, both metabolic pathways, the methionine (gene labeled SAM and MET6) and cysteine (gene labeled SAH and CYS) were shown to produce reactive Se. Reproduced with permission from ref (122). Copyright 2018 SpringerNature.

A similar biosynthetic pathway for CdSe quantum dot production was identified in the yeast *Saccharomyces cerevisiae*.^122^ In work by Shao et al., selenite reduction was shown to use the pre-existing sulfur respiration pathways in the cell, and selenium compounds SeMet, SeCys_2_, SeCys, and SeHCys were all identified as intermediates. Importantly, the yeast were then genetically modified to block the cystathionine pathways ΔCYS3 and ΔCYS4, which resulted in significantly less CdSe production compared to the wild type. The authors also identified that SeMet was an important intermediate by demonstrating the upregulation of the first two proteins in Se-methionine metabolic pathway, SAM1 and SAM2, in the presence of cadmium chloride. Finally, CdSe production was enhanced by introducing an alternative SeMet pathway, MET6, into the yeast, increasing the bioavailability of SeMet, which can then be subsequently converted into SeCys for reaction with Cd to produce CdSe (Figure 8).

Other proteins implicated in the reduction of selenite to a reactive form of Se^2–^ include several reductases, such as superoxide reductase and cytochrome c reductase.^21,123^ In some cases, selenium reducing pathways must be carefully balanced to favor to production of CdSe over the precipitation of elemental selenium. In one example, the metal-reducing bacteria *Shewanella oneidensis* was applied to produce CdSe quantum dots.^20^ Metal reducing bacteria rely on extracellular electron transfer (EET) to reduce selenite into elemental selenium. With the introduction of cadmium chloride into solution, *S. onedidensis* produced a mixture of Se nanoparticles and CdSe. However, the authors found that when the primary membrane protein for EET, CymA, was deleted from the bacteria, CdSe was the predominantly produced material. This indicates that CdSe formation occurs in the cytoplasm through previously proposed routes such as GSH reduction, while EET favors reduction to elemental Se nanoparticles that can be exported from the periplasm. This is a key example of how uncovering the function of enzymes within the cell can produce higher purity nanomaterials such as quantum dots.

**Figure 9:**
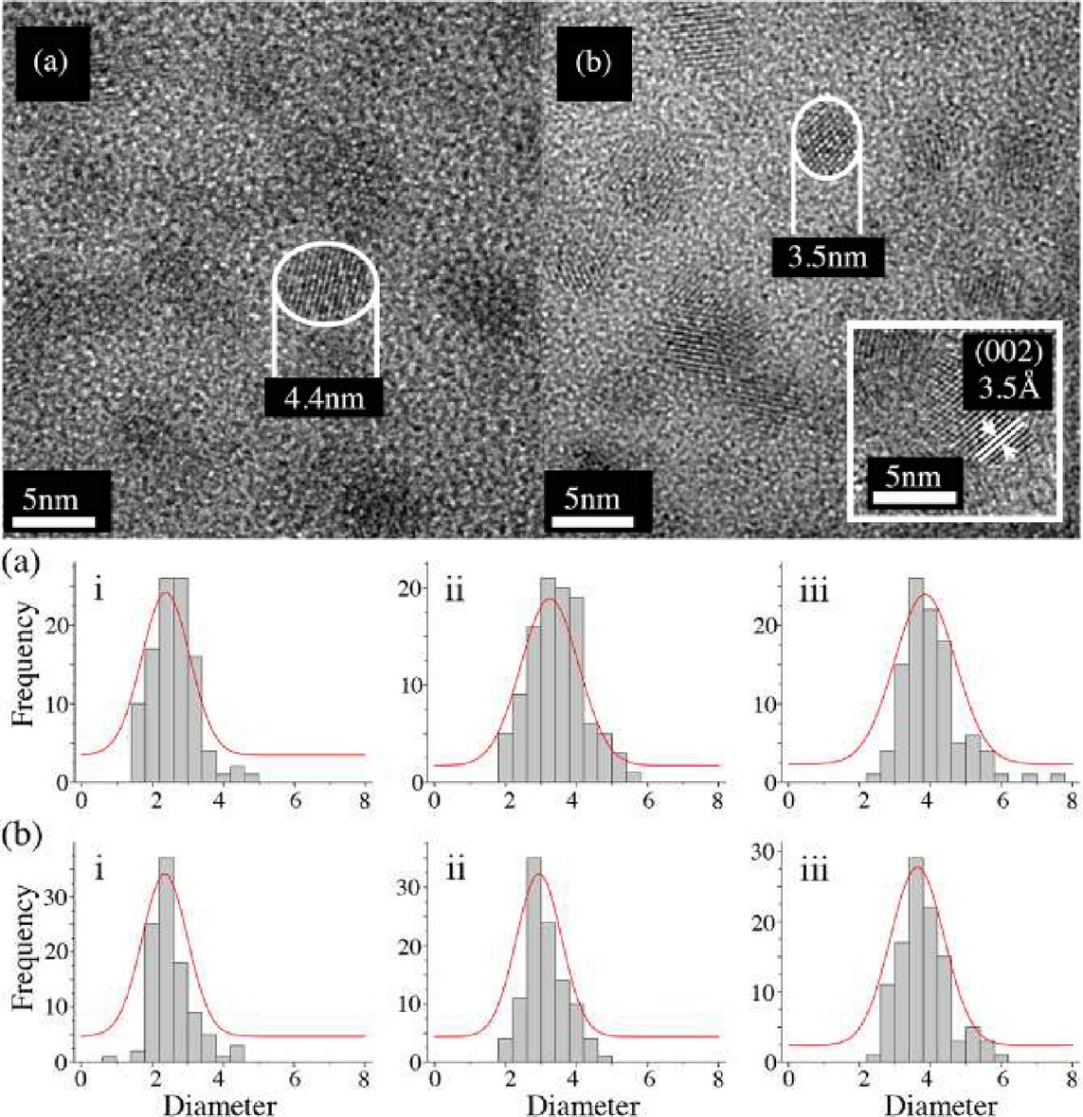
TEM imaging and calculated size distributions of a) abiotic and b) biotic CdSe nanoparticles demonstrating comparable uniformity in size even when using a biomineralization approach. Reproduced with permission from ref (124). Copyright 2013 IOP Publishing Ltd.

Work by Pearce et al. took a step toward controlling nanoparticle synthesis by separating the reduction of Se from the reaction with Cd, resulting in highly stable, more uniform distributions of CdSe quantum dots.^124^ In the work, the anaerobic bacteria *Veillonella atypica* was used to reduce selenite into Se^2–^ ions under an inert atmosphere. The ions were then filtered to remove any cells or proteins, and mixed with cadmium perchlorate and GSH to produce CdSe quantum dots. A similar approach was then used to produce ZnSe quantum dots by replacing the metal precursor. Compared to a similar abiotic synthesis, the quantum dots were highly uniform in size distribution (Figure 9). The protein identified to reduce selenite was methylmalonyl-CoA decarboxylase, a different reductase from the other studies; however, these bacteria grow under anerobic conditions, therefore the biochemical pathway has different reduction constraints.

##### 1B.2b.ii Cystathionine γ-Lyase

The above work exemplifies the importance of coupling multiple biosynthetic pathways within the cell to reduce Se while producing a reactive and bioavailable compound for CdSe production. However, the group of McIntosh et al. showed that CdSe could be produced *in vitro* by using a specific enzyme that catalyzes the turnover of a Se biointermediates to produce CdSe in a cell-free environment.^125^ Here, the precursor seleno-l-cystine was dissolved in solution under an inert atmosphere, followed by the introduction of cadmium acetate and the purified enzyme cystathionine γ-lyase. This enzyme was previously found to catalyze the production of HS- from l-cysteine.^109^ When seleno-l-cystine is present, the protein produces Se^2–^ which can then react with Cd available in solution. Because this biomineralization approach occurs completely outside of the cell and thus lacks the presence of any naturally occurring proteins or biomolecules to act as capping agents, an exogenous capping agent must be added to stabilize the quantum dots and improve their applicability to technologies such as solar cells and biotagging. The capping agent 3-mercaptopropionic acid (3-MPA) was found to stabilize CdSe quantum dots with size-controlled optical properties, and subsequent CdSe quantum dots had a high degree of crystallinity and were applied in a quantum dot sensitized solar cell.

##### 1B.2c Telluride Reactions

Telluride is chemically similar to selenium, with a slightly different electronegativity (2.1 Te and 2.55 for Se) and a difference in crystal phases at room temperature, with Te occurring only as trigonal and Se occurring in trigonal and monoclinic.^74^ Despite these minor differences, there have been no direct reports using microorganisms or proteins to produce metal Te quantum dots. While reports do exist of Te biomineralization (*vida infra*), producing CdTe is likely difficult because the reaction of Cd with Te must be coupled within the cell. We first discuss potential issues with reducing the oxyanion tellurite to the reactive form Te^2–^ that limit the biomineralization of metal telluride nanoparticles, based on the insightful review of Se and Te nanoparticle biomineralization by Kessi et al.^74^

Tellurite reduction is more difficult than selenite reduction because tellurium behaves differently in the cellular environment. Tellurium has a high affinity for thiol residues, and thus likely binds irreversibly to available sulfur groups on proteins or biomolecules, especially in microorganisms.^74^ Additionally, most studies on the toxicity response of telluride indicate that the microorganism is able to reduce the Te^2–^ to elemental Te in the periplasmic space,^126,127^ or completely shut off transport pathways, limiting the influx of ions in the first place.^128^ The lower availability of reactive Te^2–^ combined with its limited access to the cytoplasm likely prevents its incorporation into metal telluride nanoparticles via the same pathways observed for metal selenides.

There is, however, one reported instance of the complete synthesis of CdTe quantum dots using a biomineralization approach. Stürzenbaum et al. reported the synthesis of CdSe by the standard earthworm *Lumbricus rubellus* after 11 days of growth in soil containing CdCl_2_ and Na_2_TeO_3_ salts. The biosynthesis was proposed to occur via the glutathione reductase pathway, and also via reduction by NADH and glutathione itself.^129^ However, no proteins were identified and thus it is possible the biosynthetic pathway is different. Regardless, the telluride reduction via proteins remains a challenge *in vitro*, and may require multiple proteins operating simultaneously or a specific aqueous environment.^129^

### 2 Biomineralization of Metal Oxide Nanoparticles

Metal oxide nanoparticles (MoNPs) have a variety of applications in solar cells, heterogeneous catalysis, waste treatment, and more.^130−133^ As with semiconductor quantum dots, the surface area to volume ratio and size tunability of MoNPs result in unique optical, mechanical, and electronic properties;^134^ however, MoNPs have the advantage of contributing ionic and covalent bonds leading to an abundance of diverse electronic properties.^135^

**Figure 10:**
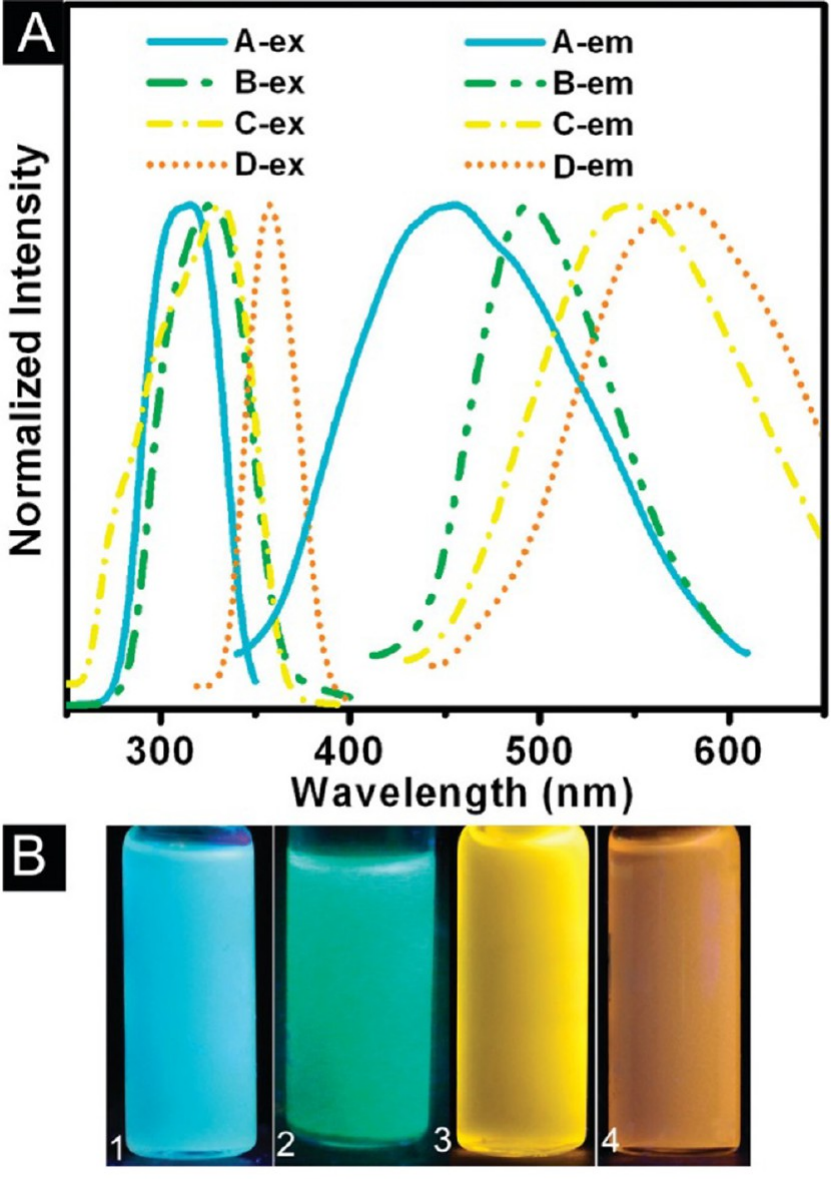
a) Luminescence from nano-ZnO, demonstrating the effect of size on optical properties with b) corresponding photo. Reproduced with permission from ref (136). Copyright 2010 American Chemical Society.

A simple example of particle size influencing properties can be seen with ZnO: bulk ZnO is white in color and does not have luminescent properties; however, nano-ZnO displays red-shifted luminescence (Figure 10).^136,137^ These changes in optical properties are often attractive for imaging and sensing applications.^134,136^ Although optical properties often change with size, each metal oxide exhibits different characteristics that cannot be predicted or described by one overarching theory. For example, the Effective Mass Theory predicts band gap as an inverse dependence on particle radius (r^–2^ or r^–1^), meaning that band gap increases as particle size decreases. This is true for Fe_2_O_3_ and CdO, and somewhat accurate for ZnO and SnO_2_; however, it does not describe the optical properties of CuO_2_, CeO_2_, and others.^138^

Similar uncertainty surrounds our understanding of MoNP mechanical properties, such as strength: the mechanical properties vary from those of bulk materials; however, they tend to be species-dependent with no obvious trends.^138^ Fu et al. describe mechanical properties as a function of size-dependence, describing a “critical size” at which mechanical properties increase dramatically.^139^ Approaching the critical size, however, the mechanical properties do not vary significantly and the critical size for each species varies.^139^ In part, the lack of discernible trends may be due to the wide variety of a given MoNP that can be produced. Very rarely do two studies produce nanoparticles of the same size that can be compared across nanoparticle species, or particles of the same species but a variety of sizes.^140−142^ This is true for both chemically and biologically synthesized particles, and an overall understanding of these size, structure, and property relationships would be improved with increased consistency. In one example, MoNPs such as ZnO, TiO_2_, and Fe_2_O_3_ have notable antibacterial properties; however, the literature does not agree on the mechanism of this activity—whether it is due to simple particle size, ion release, or even “rough” surface structure being possibilities.^142^

MoNPs are most distinguished from quantum dots and metal nanoparticles by their unique interfacial interactions, which arise due to naturally high density, an abundance of corners or edges at the interface, and the role of oxygen.^134,138,143,144^ Surface coordination, redox and acid–base properties, and oxidation state at the surface all play a role in determining nanoparticle chemical properties.^138^ These interfacial interactions tend to deviate from those seen with the bulk metal oxides, due to the important role of the surface to volume ratio and as charge effects extend throughout the material.^138^ Transition MoNP species, such as SnO_2_, CoO, CuO_2_, and CeO_2_, can be particularly attractive since they have multiple oxidation states and can therefore act as oxygen reservoirs.^145^ In the case of CeO_2_, Ce_2_O_3_ is a thermodynamically stable redox product and depending on surroundings. CeO_2_ and Ce_2_O_3_ interconversion can act to create oxygen vacancies and perform oxygen storage, facilitating a variety of applications in catalysis.^133^

For ZnO in particular, unique surface effects result in novel nanostructures such as nanosprings, nanohelices, nanoflowers, nanobows, and more.^138,146^ These novel nanostructures can result in increased surface area, porosity, and interesting oxygen vacancies.^147^ In the case of nanoflowers, these structures display increased light absorption than their traditionally shaped counterparts, which researchers have applied to photocatalytic organic dye degradation.^148^ Ultimately, the role of oxygen in MoNPs drives stronger intermolecular interactions and greater thermodynamic stability than seen in nanoparticles without oxygen, leading to their diverse and unique properties.^135,149,150^ These characteristics make MoNPs very useful in solar cells, heterogeneous catalysis, waste treatment, and other applications, thus increasing demand.^130−133^

#### 2A Chemical Synthesis of MoNPs and Desired Properties

As previously discussed in the Introduction, MoNPs can also be made via a top-down or bottom-up approach. Due to the vastly differing properties associated with different particle size, polydispersity is an important parameter. Associated ligands are generally fairly simple to interchange and vary based on end-use applications. Finally, the sustainability of nanoparticle synthesis is becoming increasingly important with the demand for green approaches to science and technology.

The most common top-down method for MoNP synthesis takes a mechanochemical approach, starting with bead milling of reactive precursors.^151^ Mechanochemical synthesis is considered to be “green” since it avoids the use of organic solvents and high temperatures. In fact, the mechanical energy input during milling facilitates the chemical reaction from precursor to metal oxide. However, this process offers limited size control and requires downstream separation from milling beads.

Another “green” synthesis technique is the anodizing wire technique. Here, at room temperature and in aqueous conditions, energy flow through a metallic wire in basic conditions produces MoNPs.^97,152^ Interestingly, this technique yields size-tunable particles with no surfactants or capping ligands;^152^ however, there are few reports of this technique being used.^153,154^ Another emerging technique is laser ablation in liquid (LAL), where an intense, focused laser beam is pulsed onto a substrate in a liquid solution of metal precursors, producing a plasma that can reach 5000 K and allow the crystallization of nanoparticles. This technique has been shown to produce copper doped SiO_2_ nanoparticles from a wafer, and will likely see future use in synthesizing ligand free metal oxide nanoparticles.^155^

Frequently used bottom-up approaches include wet chemistry techniques, such as microwave-assisted, thermal decomposition, and solvothermal synthesis. Each utilizes high temperatures and pressures along with highly reactive species and organic solvents. These techniques are reviewed thoroughly by Nikam et al. 2018.^156^ Each technique offers some degree of control over particle size; however, often the particles tend to be polydisperse. At an industrial scale, gas phase condensation is most popular. In gas phase condensation, the metal of interest is superheated to the gaseous phase, where it then interacts with gaseous oxygen to form MoNPs.^157^ Work by Patelli et al. shows dual metal oxide particle formation with this technique, highlighting the intercomplexation between the two metal species and product morphology.^158^

#### 2B Biosynthesis of MoNPs

Biological approaches to MoNP synthesis are of interest because they avoid the use of hazardous chemicals and are environmentally benign as the biomolecule or enzyme activity takes the place of energy intensive reactions used in most chemical techniques. For example, the microwave-assisted, thermal decomposition, and solvothermal synthesis techniques offer some control over particle dispersity, but require the energy-intensive use of high temperatures and pressures, where-as biological approaches occur at room-temperature and pressure. Several biogenic syntheses for MoNPs have been shown with a variety of plant extracts; however, the active compounds and mechanisms remain largely unknown.^159−163^ For the purpose of this review, we will only discuss cases where-in an active component has been identified and the mechanism is at least partially understood (examples shown in Table 3).

**Table 3:** Summary of Metal Oxide Nanoparticles Made via Templating and Catalysis^a^

Metal Nanoparticles							
Templating				Catalysis			
Protein	Nanomaterial	Size	Reference	Protein	Nanomaterial	Size	Reference
Magnetite	Iron oxide	50 nm	(173)	Silicatein	Silica	Particle size details not reported	
Solid-binding peptides	Silica	2–5 nm	(22)		Titania	2 nm	(195)
	Titania	4 nm	(13)		Ceria	2 nm	(30)
Polyamines	Silica	Pod 380 nm	(191)		Gallium oxide	75–200 nm	(194)
	Titania	140, 400 nm	(186)		Barium Oxofluorotitanate	700 nm	(196)
	Magnesia/Germania	130 nm, 40 nm	(8)	Silaffins	Silica	500–700 nm	(207)
					Titania	15 nm	(210)
				Lysozyme	Silica	300–600 nm, 8–10 nm aggregated to ∼500 nm	(212, 9)
					Titania	20–30 nm, 10–50 nm aggregated to ∼100 nm	(212, 211)

a This is representative but not inclusive of all examples referenced in text.

##### 2B.1 Biomineralization Templating for the Production of MoNPs

##### 2B.1a Magnetite

Iron oxide, Fe_2_O_3_ and Fe_3_O_4_, is known for being an exceptionally thermodynamically stable variety of MoNP while also displaying useful magnetic and catalytic properties.^164^ These magnetic properties may be especially useful for applications in alternative energy; however, there is a need for inexpensive, monodisperse particles which is not met with current popular synthesis techniques.^130,165^

Iron oxide nanoparticles, specifically Fe_3_O_4_ is colloquially known as magnetite, and has been shown to be produced in magnetotactic bacteria.^166^ In magnetotactic bacteria, this synthesis is localized to special organelles known as magnetosomes. Other cases of magnetite formation in chiton and the human brain have also been described, although mineralization in these cases seems to be on a relatively limited scale.^167,168^

In magnetosomes, the Mam (magnetosome membrane associated) and Mms (magnetosome membrane specific) proteins have been a central focus for magnetite biomineralization *in vitro*; however, no singular protein appears to be capable of this biomineralization alone.^169^ Mam proteins are colocalized to magnetosomes; however, they are not necessarily all complexed with one another.^169,170^ MamE, MamO, and MamP are all necessary contributors to biomineralization, with individual knockouts showing decreased or abolished activity.^171−174^

Several Mam proteins together template particle formation, including MamC, MamE, MamF, MamG, and MamO.^166,173^ MamE and MamO have sequence similarity to serine proteases; however, Hershey et al. show that magnetite biomineralization is not mediated by the enzymatic active site, but a dihistidine motif. The dihistidine motif coordinates with a single metal atom, which facilitates the movement of iron atoms into the magnetite lattice structure.^173^ Coordination of iron via intermolecular interactions with the imidazole side chain in the dihistidine motif is analogous to the general templating activity described previously.

MamP has also been examined as a key player in this biomineralization, with an acidic crucible-shaped pocket identified as oxidizing iron II to ferrihydrite (Fe_5_O_8_H), which is then transformed to magnetite (Fe_3_O_4_) with the gradual addition of more iron II.^174^ The acidic amino acids within the pocket seem to coordinate with Fe, modulating the redox activity of this reaction. Alanine scanning mutagenesis with the acidic residues illustrates diminished biomineralization activity, highlighting the importance of the acidic side chains for magnetite templating.^174^

Both MamE/MamO and MamP templating is dependent on the atomic interactions between Fe and the imidazole (H) or acidic side chains within the active site. Magnetite formation is ultimately driven by supersaturation and coprecipitation, then crystallinity and magnetic properties are induced via templating with these amino acids.

**Figure 11:**
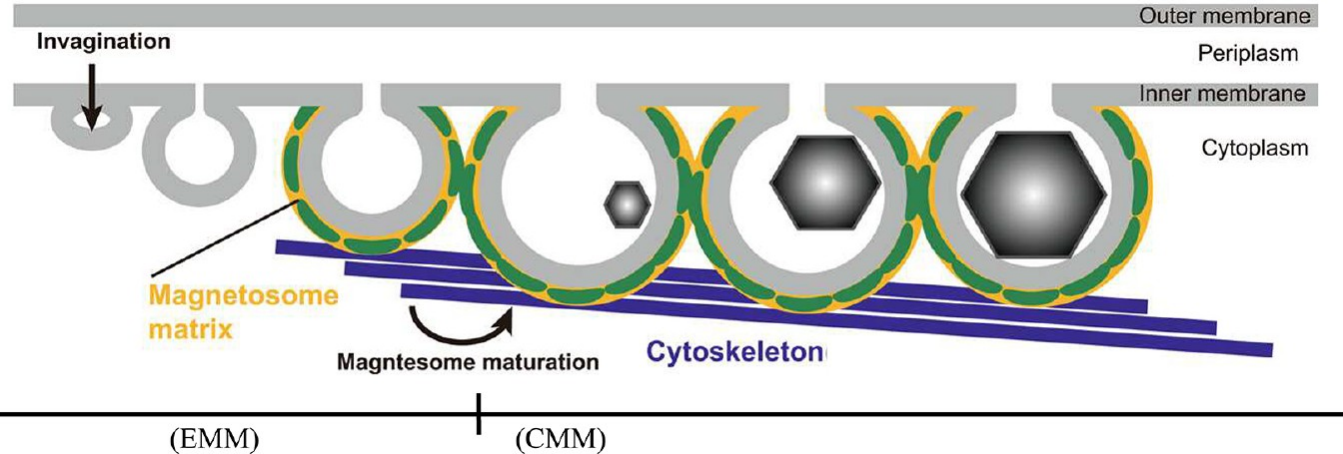
Growth of empty magnetosome membranes followed by crystal nucleation and growth in crystal-containing magnetosome membranes shown. Growth stages of invaginated magnetosome membranes as empty magnetosome membranes (EMM) and crystal-containing magnetosome membranes (CMM). Reproduced with permission from ref (169). Copyright 2023 John Wiley & Sons Books.

Templating here is not limited to the interaction of individual amino acids with redox active iron: magnetite formation is also mediated by the membrane encapsulation of growing particles. Within membrane encapsulation, the size of invaginated membrane (Figure 11) seems to play a critical role in reaching sufficiently high concentrations for nucleation, then guiding subsequent particle growth to accompany membrane growth. Figure 11, from Taoka et al., illustrates the genes and proteins involved in membrane growth, highlighting the growth of empty magnetosome membranes, then crystal-containing magnetosome membranes.^169^ A cohesive examination of MamE, MamO, MamM, and MamP by Wan et al. reveals the corresponding inactivity to individual gene knockouts, where-in MamE knockout results in inhibited crystal maturation (size limited to 30 nm), MamO and MamM knockouts individually result in empty magnetosome membranes, and MamP knockout results in smaller crystals, likely due to steric hindrance associated with less membrane growth. Accordingly, MamO and MamP appear crucial for particle nucleation, while MamE and MamP seem to regulate particle growth.^171^

Altogether the Mam proteins and gene circuit template mineralization by controlling iron precursor flux, orientation, and size. In order to effectively leverage this process for MoNP synthesis, this biological system needs to remain intact for magnetosome production to occur; however, changes in iron flux or within the gene circuit could be used to modulate particle size. Additionally, since magnetotactic bacteria growth in the laboratory is difficult,^175^ it would be worthwhile to consider a recombinant translation of these genes and proteins from magnetotactic bacteria into a higher producing species in order to potentially increase production yields.

##### 2B.1b Solid-Binding Peptides

Solid-binding peptides (SBPs) are frequently used to induce the formation and precipitation of crystalline silica and titania. Car9 (DSARGFKKPGKR), R5 (SSKKSGSYSGSKGSKRRIL), and titania-binding peptides (Ti-1 QPYLFATDSLIK and Ti-2 GHTHYHAVRTQT) have been identified as potential biotechnology tools for silica and titania synthesis.^13,22,176,177^

**Figure 12:**
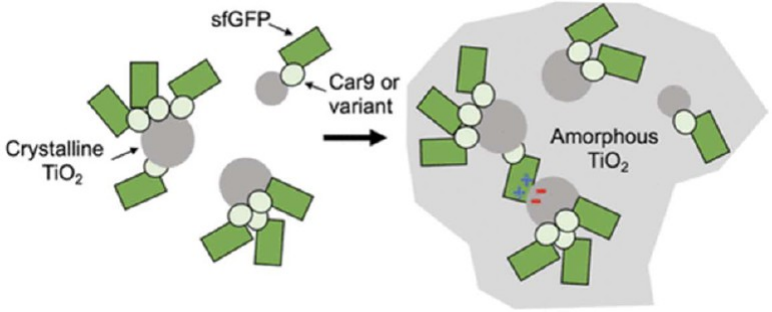
Schematic with Car9 sfGFP fusion induced biomineralization then precipitation of titania. Reproduced with permission from ref (178). Copyright 2020 American Chemical Society.

Hellner et al. compared the biomineralization of Car9 and R5 in order to examine the mechanism dependence of sequence and tertiary structure.^178^ In this work, a Car9-sfGFP conjugate induced precipitation of titania precursor, with the SBP incorporated in the final nanocrystalline product (Figure 12). An R5-sfGFP conjugate did not exhibit any notable activity.^22^ Further examination of Car9 with surface plasmon resonance and molecular dynamics simulations highlights the role of K and R residues for surface-binding interactions with the silica.^178^ Previous work by Lutz in 2017 with sum-frequency generation spectroscopy and solid-state NMR shows the entire sequence of R5 interacts with silica, with associated conformational changes throughout the biomineralization process.^177^ Notably, the R5 sequence is composed of 40% K and R residues. Lysine was examined previously by Lechner et al. and determined to be necessary for the polycondensation, and K and R residues are thought to contribute to biomineralization via their cationic head groups.^176^ This hypothesis is further supported by previous work from Nonoyama, et al. which postulates that the cationic amino group on K facilitates nucleation with a metal.^179^ Further studies have been conducted that corroborate the importance of K and R residues; however, the exact role and extent of each remains unclear.^180,181^ SBPs are described in further detail in a 2021 review by Pushpavanam et al.^182^

##### 2B.1c Polyamines

In diatoms, long-chain polyamines are found closely associated with other biomineralization peptides and proteins.^183−185^ These polyamines are generally 600–1500 Da linear chains of N and C and are often secured to individual amino acids by putrescine or putrescine-derivative linkages.^183^ Previous work by Mizutani et al. highlights the role of polyamines *in silica* polycondensation, and further studies show short-chain polyamines such as spermidine and spermine modulation the formation of MoNPs.^186,187^ Many studies have been conducted to examine the mechanism by which polyamines template nanocrystal formation, showing that at least two cationic amine tails partner electrostatically with each metal acid monomers and oligomers via ionic interactions.^185,188,189^ Therefore, long-chain polyamines found associated with biomineralization in nature have a multitude of metal coordination sites that are unmatched by those of short-chain polyamines. This is supported by the lack of biomineralization seen with putrescine and cadaverine, two short-chain polyamines with only two amino groups.^186^ Therefore, this activity only carries over to short-chain polyamines that have a sufficient number of amino groups to coordinate with metals, physically bringing them close for polycondensation.^186,188,190^

The amphiphilic nature of polyamines enables phase separation and supersaturation of metals in microdroplets, a common biomineralization mechanism.^52^ Although most polyamine studies focus on silica, other elements such as titania, germania, and magnesium oxide nanoparticles have also been synthesized with this biomimetic approach.^8,186,189,191^ Notably, the importance of K and R residues as discussed with regards to solid-binding peptides combined with additional considerations of polyamine mechanism of biomineralization may lead to a deeper understanding of the relationship between these amino acids and associated activity.

##### 2B.2 Biomineralization Catalysis for the Production of MoNPs

Catalytic biomineralization of MoNPs is distinguished by the biomolecule playing a fundamental role in starting the reaction and increasing the rate of reaction as compared to a noncatalyzed conversion.^192^ The co-occurrence of catalysis and templating is known as *direct* biomineralization.^30^ Here we are only concerning ourselves with crystalline products, so referring to the reaction as catalytic just means that the molecule starts and accelerates a reaction that might otherwise occur at a much slower rate. Rather than producing nanoparticles stochastically, nanoparticle synthesis is mediated by a protein catalyst, otherwise known as an enzyme.

##### 2B.2a Silicatein

Silicatein is an enzyme from marine sponges that converts environmental silica species to silica oxide nanoparticles that form the complex microstructures of the sponge exoskeleton.^193^ Further studies have shown silicatein acts via direct biomineralization with other inorganics to form titania, ceria, gallium, and barium oxides,^30,194−196^ highlighting a wide range of materials synthesis directions.

**Figure 13:**
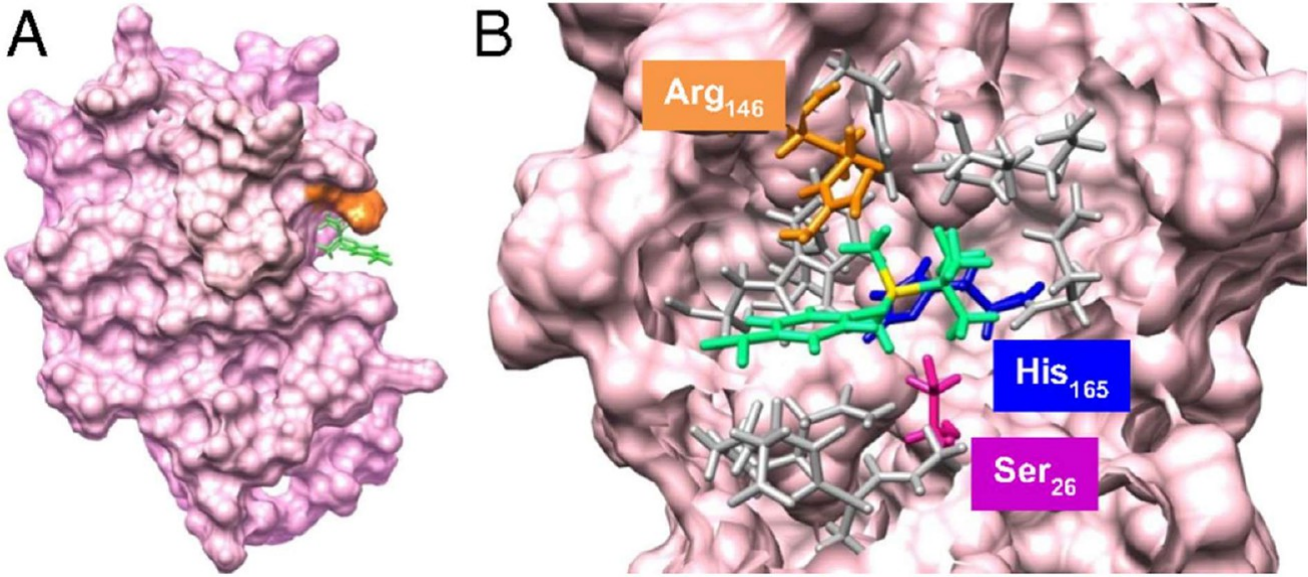
Molecular dynamics simulation of silicatein binding with substrate. A. Whole enzyme with catalytic triad shown in orange, substrate shown in green. B. Putative catalytic triad residues and substrate are highlighted to show possible mechanism of biomineralization. Figure reproduced from ref (198) under Creative Commons license for noncommercial use.

Many molecules are involved in biomineralization *in vivo*; however, silicatein-α alone is capable of producing crystalline silica *in vitro*.^197^ Based on sequence homology with protease Cathepsin L, a putative catalytic triad motif has been identified and is postulated to perform the hydrolysis and condensation reactions that transform silicon precursors to silica (Figure 13).^198,199^ This mechanism is somewhat controversial, however, as many studies have found conflicting results, which are further confounded by autohydrolysis of silicilic acid precursors.^198−203^ Furthermore, a similar catalytic triad motif was identified in magnetite biomineralization, when probed researchers found that the catalytic triad was not functionally active for biomineralization.^173^ Since magnetotactic bacteria and marine sponges are inherently different, the coincidence of catalytic triad motifs between these biomineralization proteins is interesting, and could lead to evolutionary biomineralization studies targeting a common driver in the development of this trait.

Although the precise catalytic site has not been identified, there are significantly increased rates of biomineralization with silicatein than without, which seem to follow Michaelis–Menten kinetics,^198,204^ thus entailing that silicatein is catalyzing the conversion of precursor to metal oxide. Silicatein notably increases the rate of mineralization as compared to a no-silicatein condition (*k*_cat_ = 988 min^–1^ vs 611 min^–1^).^198^ However, these reaction kinetics are still rather slow, and it should be noted that silica precursor species are sparingly soluble, thus making experiments difficult. Recent work by Vigil et al. suggests that examining silicatein biomineralization with ceria may be an advantageous approach to kinetics studies in order to avoid the confounding effects of TEOS autohydrolysis in silica production.^205^ In order to compensate for the slow kinetics, researchers have strived to increase enzyme solubility via the addition of many different solubility fusion tags, including Trigger-Factor, glutathione, maltose binding protein, and Pro-S2.^202,204,206^ No impactful increases in biomineralization activity have been associated with attempts to increase solubility, in fact, some work suggests that even aggregated silicatein is biomineralization active.^205^

In order to make silicatein more effective for MoNP biosynthesis, enzyme kinetics should be a target of study. Bawazer et al. took a pseudoevolutionary approach via DNA shuffling to generate a library of biomineralization mutants, identifying two mutants with increased binding activity.^14^ However, genetic engineering of silicatein has been limited to fusion tags since then. Furthermore, since silicatein works with several substrates, and can thus produce many different species of MoNPs, it is an attractive candidate for biological synthesis; however, increasing reaction kinetics is paramount to make this approach economically enticing.

##### 2B.2b Silaffin

Silaffin, another protein originating from diatoms, has also been noted for it is catalytic biomineralization activity. Originally isolated from *C. fusiformis*, silaffins have since been found with silicateins in other marine sponges.^15,207^*In vivo*, silaffins have extensive post-translational modifications with the addition of polyamino groups and various glycosylation, phosphorylation, and sulfations that lead to the generation of crystalline biominerals.^208^*In vitro* silaffin has been shown to mineralize crystalline silica and titania; however, there are many reports of amorphous biomineralization as well.^207,209^ Sumper et al. showed improved biomineralization when silaffin and long-chain polyamines were used in tandem, reinforcing their co-occurrence in nature.^183^ Although silaffin particle production has been somewhat variable *in vitro*, surface-anchored silaffin retains biomineralization activity with crystalline outputs in spite of secondary structure conformational changes. Kharlampieva et al. note that random coil and β sheet secondary structures influence biomineralized titania particle size.^210^ This suggests that the individual functionalities associated with amino- and hydroxyl- amino acids are sufficiently prevalent to accommodate structural changes within the protein while preserving biomineralization ability. Furthermore, this finding supports the lack of a specific recognized catalytic motif identified in silicatein.

##### 2B.2c Lysozyme

Lysozyme has been shown to catalyze the formation of silica, titania, and other oxide nanoparticles, increasing the production of these particles by several fold.^211,212^ Ding et al. also thoroughly review other functional nanomaterials that can be synthesized with lysozyme. For metal oxide nanoparticles, lysozyme interactions appear to be electrostatic, with hydrogen bonding facilitating coordination with the metal ion.^213^ Lysozyme mediates the transformation from TiBALDH to anatase nanotitania in a concentration dependent matter.^9^ This concentration-dependence is initially linear; however, it does reach a point of protein saturation with precursor. These results are consistent with Michaelis–Menten enzyme kinetics, as observed with silicatein. One notable limitation of lysozyme-mediated biomineralization is enzyme incorporation within the nanomaterial, reportedly increasing the degree of postproduction purification required. A recent examination of lysozyme biomineralization activity by Stawski et al. with small-angle X-ray scattering shows aggregation of enzyme and metal molecules consistent with formation by a diffusion-limited particle cluster aggregation mechanism, or supersaturation due to phase separation.^214^ Lysozyme will also be discussed in the following section—this is indicative of enzyme–substrate flexibility. However, it should raise some concern regarding enzyme specificity within a substrate mixture.

### 3 Biomineralization of Metal Nanoparticles

Elemental metal nanoparticles have diverse applications in medicine and catalysis. In medicine, they are frequently looked to for their antimicrobial properties—as elemental metals, atomic leaching is very toxic, which is a characteristic advantageous for antimicrobial applications but potentially harmful upon release to the environment.^215−218^ In catalysis, elemental metal particles are frequently used for their electrocatalytic activity, with applications ranging from CO_2_ reduction to incorporation in fuel cells.^219−221^ For these applications, elemental metal nanoparticles are better than metal-oxide nanoparticles due to their unique catalytic abilities.

Noble metal nanoparticles such as Au, Ag, Pt, and Pd are known for their resistivity to oxidation and corrosion, which contributes to their relative stability as elemental metal nanoparticles. In contrast, there are very few cases of stable transition metal nanoparticles such as Fe, Cu, Ni, and Co due to their readiness to oxidize to more stable metal oxides.^219^ Although there is an abundance of metal nanoparticle biosynthesis reports, many are “proof-of-concept” without significant detail or very specifically focus on one metal substrate. In order for any of these approaches or techniques to become widely productive at a large scale, it is likely that significant focus will need to be devoted to a mechanistic understanding of the process and subsequent optimization for these techniques.

#### 3A Chemical Synthesis of Metal Nanoparticles and Desired Properties

Metal nanoparticles can be made via physical or chemical approaches, with techniques such as microwave irradiation, pulsed laser ablation, supercritical fluids, impregnation, coprecipitation, chemical vapor deposition, or electrochemical reduction, and have been reviewed thoroughly by Campelo et al. and Jamkhande et al.^219,222^ Notably, these methods often require high temperatures and pressures, and sometimes even a secondary reduction step. Furthermore, size control varies widely with each methodology. For example, the supercritical fluid approach requires reaction at 80 °C and 30 atm, then a postsynthetic reduction step at 200 °C, and produces particles of a relatively wide size distribution.^223^ In other examples, the impregnation technique requires incubation at 300 °C overnight, treatment with organic solvents, and an air-free environment, while the popular chemical deposition method often requires temperatures of 900 °C (although temperatures as low as 500 °C have been reported).^224,225^

As discussed previously in Sections 1 and 2, the key to the unique nanoregime properties stems from surface area to volume ratio. One study with Cu NPs shows a direct relationship between size and catalytic activity by evaluating NPs as small as 2 nm to 15 nm.^226^ When evaluated for catalytic activity for CO_2_ reduction, researchers saw a significant increase in catalytic activity for particles 2 nm in diameter as compared to 4 nm, then 15 nm and bulk.^226^

**Figure 14:**
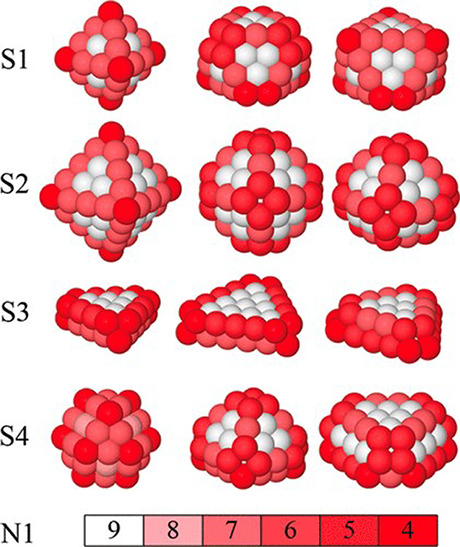
Nanoparticle shape determines surface exposure of atoms and coordination to other atoms within the particle. S# determines classification of particle shape, while N1 scale shows coordination to other atoms, with 9 denoting 9 coordination points. Reproduced with permission from ref (227). Copyright 2010 American Chemical Society.

In addition to size, another key characteristic of metal nanoparticles is particle morphology, which is influential in determining catalytic activity. In Figure 14, different shapes illustrate the different coordination of an atom to another, so for example the surface atoms on a sphere have more coordination with each other than surface atoms on a wedge.^227^ Accordingly, shapes that have surface atoms less coordinated with their neighbors are more reactive than shapes where-in each atom is heavily coordinated with others. In Figure 14, the least coordinated atoms can be visualized in white, while the most coordinated are in red. Mostafa et al. saw the greatest catalytic activity with shape 3, which had the most exposed surface area.^227^ While exposed surface area is beneficial for applications in catalysis, it also improves antimicrobial activity by increasing toxicity to biological systems.^218^

#### 3B Biosynthesis of Metal Nanoparticles

Biomineralization stands in stark contrast to abiotic synthesis routes by relying on microorganisms to template the nucleation of nanoparticles, or by reducing the precursors into metallic nanoparticles. Biomineralization also often gives some degree of size control by constraining size via steric hindrance. Future work on the biomineralization of metal nanoparticles may enable the formation of specific structures or patterning of nanoparticles onto a surface.

There are many reports of biogenic nanoparticle synthesis; however, one should be cautioned that some involve the use of harsh chemicals such as NaBH_4_ or produce nanoparticles with limited control over size or shape.^228,229^ For example, biomineralization by plants and plant extracts has been widely reported; however, details concerning mechanism and control over particle size and shape is limited.^23,230−232^ Here we will address biogenic nanoparticle synthesis via templating and catalysis, without the use of additional harsh chemicals. A summary of the proteins, nanomaterials, and relevant characteristics are shown in Table 4. The cases where NaBH_4_ is the reducing agent will not be discussed.^233^

**Table 4:** Summary of Metal Nanoparticles Made via Templating and Catalysis^a^

Metal Nanoparticles							
Templating				Catalysis			
Protein	Nanomaterial	Size	Reference	Protein	Nanomaterial	Size	Reference
Amyloid fibers	Gold microflakes	Microns	(234)	Lysozyme	Gold	18 nm	(216)
	Silver	12 nm	(235)		Silver	8–12 nm, 20 nm	(215, 252)
	Platinum	2 nm	(220)	Reductases	Gold	1 nm, 12.5 nm	(251, 16)
	Palladium	2.5 nm	(221, 240)		Silver	27 nm	(249)
Peptides	Gold	40, 3–10 nm	(238),		Gold–Silver Alloy	3 nm	(251)
	Silver	5 nm, 20 nm	(242, 239)		Selenium	100–200 nm	(258, 257)
					Tellurium nanorods	10–200 nm	(260)
					Chromium	100–200 nm	(258)
				Hydrogenases	Platinum	5 nm, 100–180 nm	(264, 263)
					Palladium	1–7 nm	(265)
				Electron Transport Proteins	Selenium	Nanorods, rosettes, nanospheres starting at 10 nm	(256)

a This is representative but not inclusive of all examples referenced in text.

##### 3B.1 Biomineralization Templating for the Production of Metal Nanoparticles

##### 3B.1a Amyloid Fibers

Amyloid fibers, resulting from the aggregation and fibrillation of β sheets, have been shown to effectively template metal nanoparticle synthesis.^234,235^ In 2015, Zhou et al. reported gold nanoparticles formed via incubation with β lactoglobulin amyloid fibrils in a concentration dependent manner. In low pH conditions, the amyloid fibrils reduce, nucleate, and stabilize chloroaurate ions to gold nanoparticles, whose shape can be mediated by varying fiber concentrations. With lower concentrations of templating agent, the authors show that growth overrides nucleation, allowing for the 2D extension of crystals to form the flat planar structures of microflakes. This work is in agreement with previous work by Fei et al. illustrating the role of silk fibroin amyloid fibrils for silver nanoparticle formation.^235^ Similar methods have been used to create platinum and palladium nanostructures as well.^220,221^ Templating nanoparticle formation with amyloid fibers is an example of liquid–liquid phase separation and saturation driving biomineralization thermodynamically.^52^ The fibers serve as nucleation sites for metal ions, which can then be reduced to their zero-valence state. Notably, this reduction can be mediated by the amyloid fibrils themselves, some nucleation peptide, or the addition of harsh chemicals such as NaBH_4_.

##### 3B.1b Peptides

Many works examine peptide templating for nanoparticle synthesis, with some activity attributed to peptides as short as 7 amino acids and others as long as 30.^236−241^ There is also some variety for the reaction conditions at which these peptides work, which can be “alone” in solution, attached to a surface or membrane, or even *in vivo* in a cellular environment.^239−242^

Early work by Brown et al. examines the biomineralization of gold nanoparticles via *E. coli* surface display of 30-mer peptides, identifying adherence and reduction as critical functions.^241^ Here, adherence refers to the binding or coordination of the peptide with the metal ion, which effectively immobilizes the ion close enough for reduction.^241^ Through peptide surface display, researchers identified two tetra-peptide motifs associated with higher rates of biomineralization: GASL and EKSL. Here, the authors note that the accelerated biomineralization leads to the long, thin crystals observed. Although GASL and EKSL within the 30-mer show higher rates of biomineralization, there is no “reduction” activity attributed to these tetrapeptides. Instead, general acid base reactions within the microenvironment surrounding these tetrapeptides begins the conversion of Au(III) to Au(0).^241^

**Figure 15:**
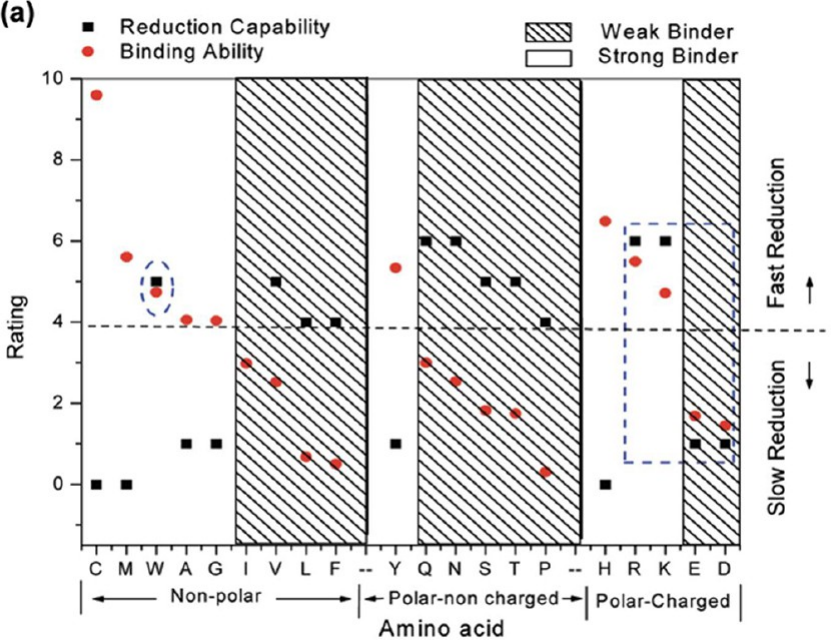
Reduction and binding strength for the 20 canonical amino acids. Reproduced with permission from ref (238). Copyright 2010 American Chemical Society.

Similarly, Tan et al. claim that the role of peptides in biomineralization is dual, ultimately balancing binding and reduction.^238^ With consideration to these properties, they evaluated the 20 canonical amino acids for each, noting that binding and reduction seem to be inversely proportional to each other, i.e. better binders are not good at reducing, and good reducers are not good binders (Figure 15). This result is consistent with a single amino acid not being enough to completely facilitate biomineralization on its own. As such, C, H, and M were identified as the best binders, with their charged sulfur and nitrogen substituted side chains complexing to metal ions. W was identified as the best reducer, with R and K alongside.^238^ Therefore, a peptide with some combination of C/H/M and W/R/K would be a promising candidate to bind and reduce metal ions. However, multiple reduction AA in a row did not generate a cumulative effect (seven W did not reduce more than two W), and alternating binding amino acids with reducing amino acids was not as productive as alternating binder, reducer, and spacer amino acid.^238^ Although the authors do not explicitly state this, alternating the functional amino acids with “spacer” amino acids likely contributes to a more favorable secondary structure and thus orientation around the substrate ion. Thus far, specific characteristics or biochemical contributions of the spacer amino acids are not well understood, although presumably less reactive side chains would be preferential to reactive ones.

In comparison to the findings of Brown et al., researchers incorporated strongly reducing amino acids between the tetrapeptides GASL and SEKL.^238,241^ The peptide SEKL-WW-GASL showed increased biomineralization compared to SEKL-GASL, suggesting that the strongly reducing W contributes positively to Au biomineralization.^238^ Additionally, these results suggest that there are some additive benefits from combining different biomineralization peptides.

Other works note the role of C/H/M for metal binding/complexation and W as a strong reducer.^237,239,240^ Tanaka et al. examined the mineralization of gold and silver nanoparticles via high-throughput screenings of peptide arrays. In their work, decapeptides with W and H frequently occurred as high-achieving biomineralization templates.^239^ In the case of silver nanoparticles, M, H, and W were in each of the top three performing peptides, along with an EE or EXE motif (AgMP1; AESEHEWEVA, AgMP2; EEPHWEEMAA, and AgMP3; PEESQEGWMA). Although the authors do not speculate the role of EE or EXE, they do note that this finding suggests different mechanisms for biomineralization for gold and silver nanoparticles.^240^

In one interesting case, decapeptide “Ge8” (SLKMPHWPHLLP), which was originally identified for it is role in mineralization of germania, along with HEPES buffer, light, and silver precursor, produced silver nanoparticles but did not show comparable activity with gold.^242^ Here, the authors speculate that photocatalytic degradation of the HEPES buffer to form hydrogen peroxide kicks off reduction, after which the peptide facilitates nucleation and growth. In many cases, templating or catalytic agents that mineralize silver also seem to mineralize gold, and vice versa; however, this is not the case with Ge8. While silver mineralization with Ge8 yielded 4.1 ± 0.9 nm particles over a period of hours, immediate massive precipitation occurred when gold was added to the reaction solution.

To probe the role of H, M, and W amino acids in Ge8, authors performed site-directed mutagenesis of each site to A.^242^ For the H → A mutagenesis, mineralization activity remained intact but led to particles of a greater size and polydispersity (5.3 ± 1.4 nm). For M → A and W → A mutants, biomineralization activity was lost. In juxtaposition, a full scramble of the decapeptide sequence retained biomineralization activity, but produced particles of a greater size (7 ± 3 nm).^18^

Although C/H/M and W/R/K are promising for biomineralization in a “binding” and “reduction” motif, S has also been identified as a notable AA. Phage-display mediated biomineralization with VSGSSPDS, shows directed nucleation and reduction of Au to nanowires followed by production of nanowire shells with the addition of Pt.^243,244^ Lee et al. claim that Au ion binding is facilitated by the polar serine side chain engaging in acid–base catalysis to secure metal ions, as oxygen electrons from the hydroxyl side chain move to coordinate with the metal, releasing hydrogen from the OH group. In review of the peptides in this section, most also contain at least one S residue (Ge8 SLKMPHWPHLLP, GASL, SEKL, AgMP1; AESEHEWEVA, AgMP2; EEPHWEEMAA, and AgMP3; PEESQEGWMA).^239,241,242^

Not only are peptides useful to mediate nanostructure formation, they can also be used to guide the shapes of NPs produced which is especially beneficial when considering catalytic applications. Work by Chiu et al. illustrates heptamer SSFPQPN is able to guide platinum NP growth through preferential binding to the {111} face.^236^ Ruan et al. examine the role of peptide sequence in morphology control further with several heptamer variants, producing platinum NP cubes, tetrahedra, and octahedra.^245^ It should be noted that many more examples of peptide templating for nanoparticle formation have been recorded but were not discussed here due to the inclusion of harsh chemicals such as NaBH_4_ and NaOH.^246−248^

##### 3B.2 Biomineralization Catalysis for the Production of Metal Nanoparticles

In the case of metal nanoparticles, catalysis refers to catalyzing the reduction of particles that drives supersaturation and nucleation. Several enzymes have been reported to mediate these reactions, such as lysozyme, reductases, and hydrogenases.^215,249−251^

##### 3B.2a Lysozyme

Addressed previously in our discussion of metal oxide biosynthesis (Section 2B), lysozyme catalyzes the formation of gold and silver nanoparticles. In the case of silver, Eby et al. claim that lysozyme acts as both the reducing and nucleating agent. The enzyme’s cationic and amphiphilic properties also facilitate the capping of the nanoparticles produced. As a catalytic and capping agent, the proportion of lysozyme to silver precursor acutely influences particle size, with an increased lysozyme to precursor ratio generating smaller particles.^215^ An increased lysozyme amount leads to an increased reduction and nucleation of silver, rather than limited nucleation followed by moderated particle growth. Furthermore, post nucleation, the relatively high amount of lysozyme is then available for nanoparticle capping, limiting any particle growth due to Ostwald ripening.^215^ Interestingly, Eby et al. report that the native hydrolytic activity of lysozyme remains intact, suggesting that while lysozyme functions catalytically here, this activity is separate from its hydrolytic activity.^215^ In contrast, Rey et al. report that the conformational changes of lysozyme following nanoparticle capping prevents native enzyme activity.^252^ These differences are likely due to the addition of photolytic additive benzoin I-2959 present in Rey et al.’s work, which reportedly aided in reduction and nucleation; however, it notably influenced the final colloidal particle.^252^

**Figure 16:**
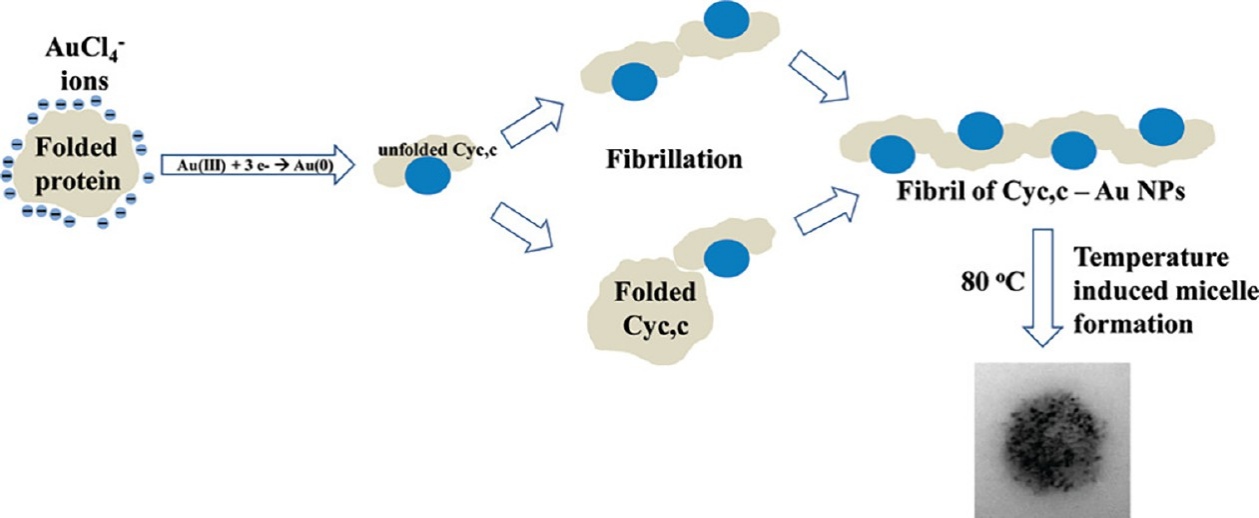
Suggested mechanisms for Au nanoparticle formation with lysozyme and cytochrome C. Temperature and protein structure may play key roles in nanoparticle formation. Reproduced with permission from ref (250). Copyright 2010 American Chemical Society.

Although lysozyme acts catalytically in metal nanoparticle production, some works suggest that the secondary structure and even identity of the protein is relatively unimportant compared to the accessibility to key amino acid residues.^216,250^ To assess the role of conformationally intact lysozyme as compared to denatured lysozyme, Bakshi et al. evaluated the formation of Au nanoparticles at 40 and 80 °C.^250^ With this comparison, the researchers show increased/faster nanoparticle formation at 80 °C than at 40 °C, which they attribute to the additional exposure of cysteine residues due to denaturation and disruption of disulfide bridges (Figure 16). In contrast to this work, Kumar et al. posit that the biomineralization activity of lysozyme is mediated by accessibility to Y and W residues.^216^ In the production of gold and silver nanoparticles, the authors claim that complexation of Y is paramount, and note the secondary complexation of metal ions with D, E, H, C, K, M enables a conformational change of the protein, wherein W comes into proximity with the metal ion and acts as the reducer. To highlight the role of Y in the biomineralization of silver, n-acetyl-imidazole was used to block the phenoxy ring of tyrosine, followed by a loss of biomineralization activity.^216^ Upon the denaturation of acetylated tyrosine, some biomineralization activity was recovered, which researchers attribute to the increased accessibility of W, as well as possible deacetylation of Y residues. Furthermore, in this work Kumar et al. argue that accessibility of reducing residues Y and W mediates particle size, showing that the lysozyme in native conformation produces Au nanoparticles of 18 nm, whereas heat denatured lysozyme produces Au nanoparticles of approximately twice the size.^216^

##### 3B.2b Reductases

Many works have shown the reduction of metal ions to metal nanoparticles with contributors of the glucose reduction pathway, including glutathione (GSH), nicotinamide adenine dinucleotide phosphate (NADP), and glutathione reductase. Both GSH and NADPH (reduced NADP) can act as weak reducing agents. With GSH, NADPH, and glutathione reductase *in vivo* it is difficult to decouple the activity of one from another in order to identify the “true” reducing agent for biomineralization. Accordingly, there are conflicting reports concerning GSH, NADPH, and glutathione reductase which we will discuss below.

Cui et al. show that glutathione (GSH) and NADPH reduce HAuCl_4_ to gold nanoparticles in a controllable manner.^16^ First, GSH reduces Au(II) to Au(I), forming a glutathione–Au(I) complex, which is reducible by the addition of NADPH. Furthermore, this research shows particle size tunability via the modulation of the NADPH concentration: more NADPH yields smaller particles than cases with less NADPH yielding larger particles.^16^ This finding supports the role of NADPH as the reducing and nucleating agent, as increased rates of nucleation have been shown to produce smaller particle sizes. The authors comment that although glutathione reductase is able to speed up the reaction, NADPH and GSH are able to facilitate this reaction without glutathione reductase.^16^

As a weak reducing agent, reactions with NADPH are slow and controlled.^251^ The slow nature of this process means that templating is able to play a stronger role in particle growth, resulting in crystalline rather than amorphous nanoparticle formation. Furthermore, the role of glutathione as a capping agent functions to aid in particle size control.^16,251^ In addition, Zhang et al. show the capability of NADPH as a solo reducer, with the NADPH-mediated synthesis of Au–Ag alloyed nanoparticles, which functions to highlight the ability of NADPH for the biomineralization of silver as well as gold.^251^

**Figure 17:**
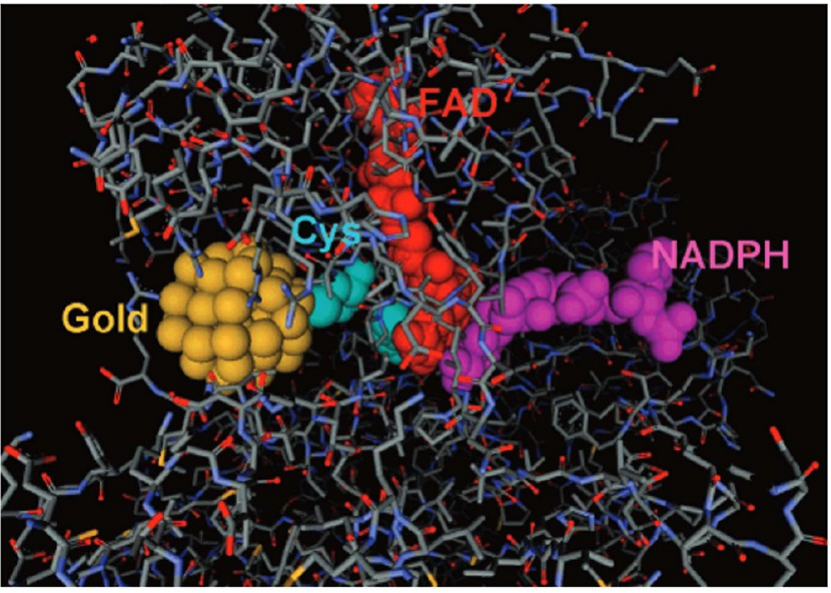
Glutathione reductase, NADPH, gold, and cofactor FAD. In this space-filling model, gold is shown binding to C42 within the active site. Reproduced with permission from ref (253). Copyright 2008 American Chemical Society.

In another case, Scott et al. show biomineralization of Au with glutathione reductase and NADPH, but not GSH.^253^ Here, the authors produced glutathione reductase recombinantly with *E. coli* and then added the Au precursor and NADPH, which mediated the formation of 2.1 nm gold nanoparticles. With glutathione reductase as the catalyst, small Au clusters are formed upon the binding of Au ions to C42 within the active site of the protein as shown in Figure 17. As the clusters increased in size, the authors observed an increase in dimerization of glutathione reductase, possibly due to cocoordination of C42 from two separate glutathione reductase particles to the same gold cluster.^253^

**Figure 18:**
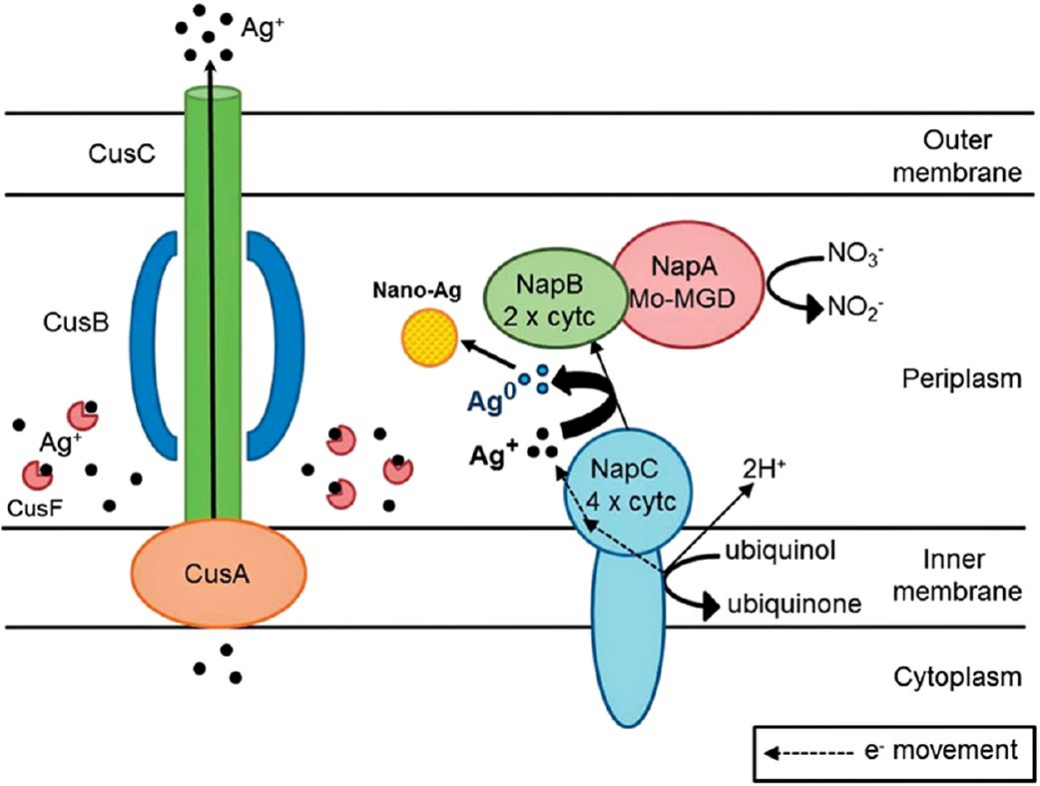
NapC catalyzes reduction of Ag for the biomineralization of Ag nanoparticles. Reproduced from ref (249). Copyright 2014 Royal Society of Chemistry.

Although there are many studies surrounding GSH, NADPH, and glutathione reductase, other reductases also exhibit biomineralization activity. NapC (nitrate reductase c-type cytochrome subunit) is a periplasmic enzyme found in environmental microbes such as *Shewanella oneidensis* and *Geobacter sulfurreducens*.^254^ In its native environment, this enzyme plays a role in reducing metal ion species so they can function as electron acceptors for the cell’s metabolic processes. Generally, these metal species are iron and manganese, producing iron and manganese oxide.^254^ Lin et al. evaluated the role of NapC in biomineralization via recombinant expression in *E. coli*, by creating a NapC knockout then later reintroducing the enzyme.^249^ In the NapC+ case, biomineralization occurred under anaerobic conditions, but not in aerobic conditions. In aerobic conditions, the cell’s chemiosmotic efflux system evacuates the metal before it can be reduced. In anaerobic conditions without NapC, no biomineralization occurs either; however, when NapC is reintroduced biomineralization activity is partially recovered. Accordingly, researchers determined that NapC is responsible for catalyzing the reduction of a silver precursor to Ag(0) (Figure 18); however, as a whole cell system with biomineralization *in vivo*, reaction control is limited, as exhibited by the production of particles of 5–70 nm.^249^

The confusion surrounding the identity of biological reducing agents continues in the literature regarding the biomineralization of Se and Te nanoparticles. Se and Te nanoparticles are mainly values for their antimicrobial activity, but they have also been shown to act as photocatalysts.^25^ The bioproduction of elemental Se and Te occurs by the reduction of selenium and tellurium oxyanions and has been identified in a significant number of microbes.^74^ A majority of this work has been performed on the reduction of selenite and tellurite as these are the most commonly observed chalcogenide contaminants in the environment, and thus many organisms have evolved detoxification mechanisms to neutralize them.^255,256^ As previously mentioned in Section 1B.2b.i, much debate remains over whether the oxyanion reduction to elemental metal requires an enzyme or can occur simply through the Painter reaction by bioavailable glutathione in the cell.^74^ However, the lack of *in vitro* examples using glutathione suggests that several sequential reactions, likely involving an enzyme, are required to produce Se or Te nanoparticles.

Two specific reductases for selenite have been found in both aerobic and anaerobic bacteria. The aerobic bacterium *Comamonas testosteroni* S44 was found to use protein SerT, a selenite reductase, to reduce selenite to elemental Se nanoparticles.^257^ In contrast to the glutathione reductases previously discussed, SerT exists in the periplasmic space of the cell and was shown to be the main driver for reduction of selenite to elemental selenium. A similar reductase named CrsF was found in the anaerobic bacteria *Alishewanellasp. WH16-1*. The protein was also found to be capable of reducing chromate to chromium nanoparticles, and had improved reduction ability when overexpressed. Intriguingly, the bacteria was found to be capable of reducing selenite and chromate when grown under aerobic conditions, despite being typically anaerobic.^258^

In addition to the selenite reductase, *C. testosteroni* also contained a separate reducing pathway specific to selenate, Se(VI).^257^ When exposed to this precursor, the pre-existing sulfur reducing pathway was shown to reduce selenate to Se nanoparticles. When the genes cysA (natively regulates transport within the cell), cysN (natively converts sulfate to APS), cysI (natively reduces sulfite), and cysB (natively regulates cysteine anabolism) were deleted, conversion to elemental selenium did not occur. The presence of an additional pathway demonstrates the wide variety of enzymes that may be capable of producing selenium nanoparticles.

Another reductase specific to chalcogenide systems is the NAD(P)H-dependent thioredoxin-disulfide reductase TrxR identified in *Bacillus sp. Y3*. The reductase was capable of simultaneously reducing selenite and tellurite and was significantly upregulated in the presence of these oxyanions. The authors also found that the sulfate reducing pathway was upregulated, again pointing to the likelihood of a multistep reduction or a simultaneous reduction pathway, aiding in the survival response to high toxicity.^259^ To further prove that TrxR was responsible for reduction, the authors overexpressed the protein and purified it using recombinant *E. coli*. Using the purified protein, selenite and tellurite were able to be reduced *in vitro* in the presence of NADPH and NADH. While the final materials were not well characterized for size or structure, this demonstration of *in vitro* reduction is a promising step toward the use of protein-mediated biomineralization of elemental chalcogenide nanomaterials.

Thus, far, the only work to demonstrate *in vitro* biomineralization of chalcogenide nanostructures was performed by Xiong et al. from Pang’s group.^260^ Remarkably, this work also clearly demonstrates how both abiotic and enzymatic reaction steps are required for Te nanorod synthesis. In this work, Te nanorods were first observed to form in living *Staphylococcus aureus*, presumably by a similar biomineralization route to selenium. The authors then mimicked the proposed biomineralization route *in vitro* by using glutathione, NADPH, and glutathione reductase to produce Te nanrods. Each biomolecule and protein were required and intermediates were clearly identified using HPLC, MS and ICP. Although introducing a strong base was required, the authors also found they could control the length of each nanorod with NaOH, which produced some TeO_3_^–^ that competed with the elemental Te monomers in solution. Such work demonstrates how a full understanding of biological processes and enzymes may lead to the use of biomolecules and proteins for the low cost, controllable synthesis of nanomaterials in the future.

##### 3B.2c Hydrogenases

Hydrogenase enzymes from sulfate-reducing bacteria have been shown to effectively biomineralize Pt and Pd ions to elemental metal nanoparticles.^261−265^*In vivo*, these hydrogenases are membrane-bound and presumably reduce ionic Pt and Pd as a detoxifying mechanism. Omajali et al. showed differential accumulation of Pd nanoparticles in different bacterial species, although hydrogenases were identified as the active site in each.^265^ Both cytoplasmic and periplasmic-bound hydrogenases contributed to the reduction of Pd ions.^261,262^ Although there are cases of both aerobic and anaerobic bacterial production of Pd(0) particles, Omajali et al. observed more extracellular particle accumulation occurring with the anaerobic species, perhaps suggesting that the low oxygen condition is favorable for the efflux of metal nanoparticles.^265^ Another study in anaerobic conditions with surface-displayed recombinant hydrogenases Hyn B and Ni-Hyd showed the production of crystalline, 5 nm Pt(0) nanoparticles.^263^

*In vitro* biomineralization with purified, recombinant hydrogenases suggests that some control is possible with the variation of hydrogen donors, metal precursor, and media conditions.^264,265^ Both H_2_ and formate are native substrates for hydrogenases; however, the oxidation of H_2_ yielded amorphous particles, while oxidation of formate gave crystalline particles.^265^ This may be due to the simple reaction kinetics of H_2_ vs formate oxidation, since formate oxidation is slower, nucleation and growth of the particle occurs more slowly.^265^ Furthermore, the valency of ionic precursor may contribute to the morphology of nanoparticles produced. Govender et al. probed enzyme activity with Pt(IV) and Pt(II) precursor compounds, noting that the Pt(II) precursor is sufficiently small to interact in the hydrogenase active site, whereas Pt(IV) is much bulkier and too large to enter the active site, and therefore coordinates on the outer surface of the enzyme.^264^ Although Pt(IV) is presumably reduced to Pt(II), which can then access the active site, Govender et al. saw the production of spherical Pt(0) nanoparticles when Pt(IV) precursor was used and rectangular or triangular Pt(0) particles when Pt(II) was the sole precursor. This result seems to contradict previous work by Riddin et al., which claims all Pt(IV) is reduced to Pt(II) prior to Pt(II) reduction.^261^ Within Riddin’s pathway, it follows that ionic precursor valency would not determine final nanoparticle morphology. Although hydrogenases are promising for the controlled production of Pt and Pd metal nanoparticles, the mechanism and tunability of this process remains unclear.

##### 3B.2d Electron Transport Proteins

While most work speculates that sulfur reducing pathways are responsible for the reduction of chemically similar chalcogenides Se and Te, The EET proteins in the bacterial cell membrane may also reduce Se or Te, preventing these toxic elements from ever entering the cell cytoplasm.^256^ The exact biosynthetic mechanism likely varies depending on the organism and toxicity response. However, EET proteins are speculated to incorporate selenite and tellurite into their respiration pathways, resulting in nanoparticles and nanorods.

## Conclusions and Looking Forward: Future Developments Needed for Improved Materials

This review presented and discussed numerous examples demonstrating protein-mediated synthesis of functional nanocrystals, taking inspiration from biomineralization in nature. Generally, biomineralization is facilitated by templating or catalysis, with peptides and proteins orchestrating the reaction of precursors to product materials. In some cases, as with reductases, the same class of protein is found to perform biomineralization across multiple organisms.^16,20,21,24,74,98,99,116,118−120,122,123,251,253−260,266^ It is also common for peptides or proteins to mediate the synthesis of several types of materials, such as CdSe quantum dots and Se nanoparticles, or as with silicatein, native product silica as well as titania, gallium, cerium, and barium oxides.^93,125,194,24,195,196,267,268^

Frequently, nanomaterial biomineralization is initially observed and performed *in vivo*; however, the few examples of *in vitro* biomineralization with purified recombinant proteins often show a higher level of control over the final nanocrystal size and shape, while also providing a deeper understanding of the nanoparticle synthesis mechanism.^109,125,267,92,253,259^ For example, in nature the cellular environment often plays a pivotal role in enabling material syntheses that require a reduction, such as for metal nanoparticles and metal chalcogenide quantum dots.^16,74,97,254^*In vitro*, there is often a relationship between the amino acid identity within the protein and its role in biomineralization. For example, C, H, and M can take a metal binding role, while W, R, and K act as reducers.^15,18,178,179,216,238,241^ These roles have been observed for both peptides and proteins.^22,41,86,173,182,243^ Future work should further investigate the active site of biomineralization and, when applicable, the relevant metabolic pathways, working to improve the enzyme specifically for biomineralization of a desired functional nanomaterial, rather than the enzyme’s evolved survival response. Such studies pave the way for making biomineralization more commercially viable and applicable for optoelectronics and catalysis, as well as other materials systems beyond those discussed here.

While nature provides numerous biomineralization pathways, the materials palette is generally restricted to metals and toxins that naturally occur in the environment. Furthermore, the state of the field relies on discovering natural proteins with known functionality. Producing other types of materials using biomineralization will require directed evolution of natural proteins, or the creation of novel, *de novo* proteins. ConK is an early example of a *de novo* protein developed specifically to perform biomineralization and demonstrates the promise of a *de novo* approach.^115^ With directed evolution and *de novo* approaches, future engineering efforts will help to expand the realm of possible materials into rare earth elements, perovskites, and other cutting edge material systems that are used in the highest performing optoelectronic and catalytic applications.
